# Effects of neuromodulation techniques on pain and depression in patients with phantom limb pain: a systematic review and meta-analysis

**DOI:** 10.3389/fneur.2025.1682650

**Published:** 2025-10-22

**Authors:** Xu-chen Tao, Shujie Ma, Jingwen Gong, Jianzhong Zhu, Jinxiang Wang, Baoyu Wu, Yan Zhu, Xinhao Liu, Lei Fang

**Affiliations:** ^1^School of Rehabilitation Science, Shanghai University of Traditional Chinese Medicine, Shanghai, China; ^2^Department of Rehabilitation Medicine, Shanghai Electric Power Hospital, Shanghai, Shanghai, China; ^3^Department of Traditional Chinese Medicine Rehabilitation, The Second Rehabilitation Hospital of Shanghai, Shanghai, China; ^4^Department of Rehabilitation Medicine, Shanghai Ruijin Hospital Gubei Branch, Shanghai Jiao Tong University School of Medicine, Shanghai, China; ^5^Department of Rehabilitation Medicine, Shanghai Zhongye Hospital, Shanghai, Shanghai, China; ^6^Department of Neurological Rehabilitation, the Second Rehabilitation Hospital of Shanghai, Shanghai, China

**Keywords:** phantom limb pain, neuromodulation techniques, review, meta-analysis, depression

## Abstract

**Objective:**

This systematic review and meta-analysis aimed to evaluate the efficacy of neuromodulation techniques in alleviating pain and depression in patients with phantom limb pain (PLP).

**Methods:**

We conducted a comprehensive search of five databases (Medline, Scopus, Embase, Cochrane Library, and Web of Science) up to March 2025, following PRISMA guidelines. Randomized controlled trials (RCTs) investigating central (e.g., rTMS, tDCS) and peripheral (e.g., TENS, NMES, PNS) neuromodulation techniques in PLP patients were included. Primary outcomes were pain reduction, measured by the Visual Analog Scale (VAS) and McGill Pain Questionnaire (MPQ), and depression, assessed using the Beck Depression Inventory (BDI) and Self-Rating Depression Scale (SDS). Data were extracted and analyzed using Review Manager and Stata, with heterogeneity assessed via the I^2^ statistic and Q test.

**Results:**

17 RCTs involving 510 patients were included. Central neuromodulation techniques, particularly rTMS and tDCS, significantly reduced pain in PLP patients [excitatory M1 rTMS: MD = −1.45, 95%CI (−2.78, −0.11), *p* = 0.03; anodal M1 tDCS: MD = −1.60, 95%CI (−2.45, 0.74), *p* = 0.0003]. tDCS with duration >15 min [I^2^ = 12%, MD = -1.91, 95%CI (−3.10, 0.72), *p* = 0.002] and rTMS>7 days treatment [MD = -4.35, 95%CI(−6.34,-2.36), *p* < 0.0001] were observed significant pooled effects. Peripheral techniques, including TENS and PNS, also showed pain relief, though with fewer studies. No significant improvement in depression.

**Conclusion:**

Neuromodulation techniques, particularly rTMS and tDCS, are effective in reducing PLP but do not significantly alleviate depression. Further large-scale RCTs with longer follow-ups are needed to confirm these findings and explore the efficacy of other neuromodulation methods.

**Systematic review registration:**

PROSPERO CRD42022314995.

## Introduction

Phantom limb pain (PLP) refers to the subjective sensation that an excised limb still exists, accompanied by varying degrees and types of pain (1, 2). PLP is one of the most significant complications after amputation, with an incidence rate of 50–80% (3–5). The onset of PLP typically occurs in the early stages after amputation. Seventy-five percent of patients may experience PLP a few days after the procedure, although some patients may begin to experience it months or even years later (6, 7).

For some patients with PLP, the pain can be alleviated to some extent through prosthetics, medication, and other treatments, but some patients continue to suffer from persistent pain. Chronic pain can significantly reduce a patient’s quality of life, affect their work ability, and, in some cases, result in the loss of social functioning. Additionally, patients may experience psychological symptoms such as depression, anxiety, speech difficulties, insomnia, obsessive-compulsive disorder, loneliness, social isolation, self-pity, and a loss of self-confidence (8, 9). The pathological mechanism of PLP is complex. Some studies suggest that PLP is a form of neuropathic pain, with a similar pathological mechanism to other types of neuropathic pain, primarily involving nerve injury and detachment. Central nervous system mechanisms propose that PLP may be linked to changes in sensory afferents, with structural and functional reorganization in the peripheral and central nervous systems playing a key role (10). Intense and persistent PLP causes considerable pain for amputees, creating an urgent need for precise and effective pain management strategies addressing the pathogenesis of PLP.

With advances in rehabilitation medicine, neuromodulation techniques have emerged as important tools in the control of acute and chronic pain, particularly for refractory chronic pain. Neuromodulation is a therapeutic technique that reversibly regulates the physiological and functional activities of the central nervous system, peripheral nerves, or the autonomic nervous system through implantable or non-implantable methods. These techniques use physical (e.g., electricity, magnetism, sound, light) or chemical methods to improve symptoms and quality of life (11).

Neuromodulation techniques can be categorized into central and peripheral nervous techniques. Peripheral techniques include transcutaneous electrical nerve stimulation (TENS), neuromuscular electrical stimulation (NMES), and peripheral nerve stimulation (PNS). These methods alleviate pain by activating Aβ fibers, which conduct coarse tactile sensations, while inhibiting Aδ and C fibers, thus reducing central sensitization and hyperalgesia (12, 13). Central nervous techniques, such as transcranial magnetic stimulation (TMS) and transcranial direct current stimulation (tDCS), regulate brain bioelectric activity, cerebral blood flow, and metabolism through electromagnetic signals, adjusting cortical excitability (14) and intervening in long-term synaptic plasticity (15) to alleviate pain.

Neuromodulation techniques hold significant potential in the treatment of PLP. However, there is no unified approach regarding the selection of techniques, timing of treatment interventions, or the setting of treatment parameters. This study systematically reviews the evidence on the efficacy of neuromodulation techniques, providing data on intensity, duration, frequency, and other relevant parameters to inform future research and clinical applications for the treatment of PLP.

## Methods

### Search strategy and selection criteria

The protocol for this study was registered with PROSPERO (number CRD42022314995) and followed the Preferred Reporting Items for Systematic Reviews and Meta-Analyses (PRISMA) guidelines (16, 17). Researchers conducted a comprehensive search of the Medline, Scopus, Embase, Cochrane Library, and Web of Science databases for studies published up until March 2025 (16). The search terms used were (1) neuromodulation techniques; (2) PLP; (3) random or allocation. Literature screening strategies are detailed in the Supplementary materials. The method of combining MeSH terms with free words was employed, with repeated preliminary examinations supplemented by manual retrieval and reference tracking.

Studies were included if they met the following criteria: (1) Type of study: Randomized controlled trials (RCTs) of neuromodulation in patients with PLP. (2) Population: Participants (≥18 years old) diagnosed with PLP who were in the non-acute stage. Gender, race, and nationality were not restricted. (3) Intervention: Central neuromodulation techniques (e.g., rTMS, tDCS) or peripheral neuromodulation techniques (e.g., TENS, NMES, PNS). (4) Outcomes: Primary outcomes included pain, measured using the visual analog scale (VAS), McGill Pain Questionnaire (MPQ).

Studies were excluded if they met any of the following criteria: (1) non-RCTs.

(2) Duplicated publications (same treatment discussed in multiple papers from the same clinical trial); (3) Missing required outcome measures or failure to report data necessary for meta-analysis (e.g., means, standard deviations); (4)Only abstracts available with no full text accessible through any channels;

### Data extraction

Two researchers ((blind*)) independently conducted the literature search. The titles, abstracts, and keywords of all studies were screened based on the established criteria. Afterward, the full texts of eligible studies were reviewed, and those that did not meet the requirements were excluded. Data was extracted from each study, including the first author, sample size, mean age, intervention details, stimulation intensity, stimulation location, and outcome measures. If data was incomplete, the authors were contacted to obtain the necessary information. If the data was not provided, the study was excluded. Discrepancies were resolved through discussion with a third researcher ((blind*)) until consensus was reached.

### Risk of bias assessment

The risk of bias for each included study was assessed using the Cochrane RCT bias risk evaluation criteria by two researchers (blind*)independently. Seven domains of bias were evaluated: (1) Random sequence generation; (2) Allocation concealment; (3) Blinding of participants and personnel; (4) Blinding of outcome assessment; (5) Incomplete outcome data; (6) Selective reporting; (7) Other sources of bias. Each domain was rated as “low bias risk,” “high bias risk,” or “unclear bias risk.” Discrepancies in evaluations were resolved through discussion with a third researcher (18) (blind*).

### Statistical analysis and data synthesis

Statistical analyses were conducted using Review Manager (RevMan, V.5.4.1) and Stata version 14.0. The I^2^ statistic and Q test were used to assess the heterogeneity of trial results and to construct pooled estimates of effect. Low heterogeneity was considered if I^2^ < 40% (19). The random-effects model (REM) was used due to expected heterogeneity across studies. Continuous variables were pooled and presented as mean differences (MDs) with 95% confidence intervals (CIs) or standard MDs (SMDs) with 95% CIs. A *p*-value < 0.05 was considered statistically significant. Publication bias was assessed using funnel plots (20) and the Egger regression test, when there were more than 10 studies in each meta-analysis.

### Sensitivity analysis

A sensitivity analysis was performed to evaluate the robustness of the review findings. One study at a time was removed, and the remaining studies were analyzed to determine whether the results were significantly affected by any single study.

### Missing data management

If the primary outcome data (e.g., VAS) was unclear, the authors were contacted for clarification. Additionally, Web Plot Digitizer version 4.5 was used to extract data from relevant graphs. If the target data could not be retrieved, the study was excluded.

## Results

### Eligible studies

A total of 3,045 articles were retrieved from five databases. After screening titles and abstracts, 879 duplicates were removed, and 2,110 articles were excluded. Seventeen RCTs were selected based on full-text screening from 59 potentially relevant studies (Supplementary materials 3, 4).

### Study characteristics

Seventeen RCTs were included in this study, with research conducted between 1991 and 2025. A total of 510 patients with PLP from various regions were included. Four rTMS studies (21–24) used 1 Hz, 10 Hz, and 20 Hz treatment intensities, targeting the primary motor cortex (M1) at different treatment periods. Six tDCS studies (25–30) used current intensities of 1, 1.5, and 2 mA with varying treatment times and locations. Four TENS studies (31–34) targeted the outer ear, pain site, or contralateral limb. One NMES study (35) targeted quadriceps muscles of both legs. Two PNS studies (36, 37) targeted the femoral and sciatic nerves with percutaneous PNS leads under ultrasound guidance. VAS, MPQ, Beck Depression Inventory (BDI), Self-Rating Depression Scale (SDS) and Hamilton Depression Scale (HAMD) were used to evaluate the effects of neuromodulation techniques on PLP patients (Supplementary material 4.3).

### Quality assessment

The quality of the studies in the seventeen included RCTs was assessed using the Cochrane Collaboration Network’s risk of bias evaluation criteria. Twelve of the RCTs (22–24, 26, 27, 29–31, 33, 35, 36, 38) used random assignment, and ten (21, 22, 24–29, 31, 32) described the method of concealed random assignment. Eight RCTs (21–24, 26, 32, 35, 37) were conducted in a blinded fashion for subjects and treatment protocol implementers. Nine RCTs (21, 22, 25–27, 29, 32, 35, 37) blinded the experimental outcome measures. Therefore, the overall quality was good (Supplementary material 4.2).

### Meta-analysis results

#### VAS

A total of 14 RCTs (21–30, 32, 33, 36, 37) with 387 patients were included in this study. Statistical heterogeneity was observed between studies by the X^2^ test (*p* = 0.05, I^2^ = 42%), and meta-analysis was performed using REM. Neuromodulation techniques were significantly superior in improving the VAS index in patients with PLP compared with the control group, with a statistically significant difference [MD = -1.61, 95% CI (−2.36, −0.86), *p* < 0.0001]. Funnel plots were performed for the VAS subgroup, showing no asymmetry. The Egger’s regression test (*p* = 0.338) did not detect significant small study effects (Supplementary Figure 5.1).

#### MPQ

Three RCTs (32, 35, 36) with 123 patients were included in this study. There was no statistical heterogeneity between studies by the X^2^ test (*p* = 0.77, I^2^ = 0%), and meta-analysis was performed using REM. Neuromodulation techniques relieved pain in PLP patients based on the MPQ index, with a statistically significant difference between groups [MD = -4.59, 95% CI (−8.12, −1.06), *p* = 0.01]. However, no follow-up analysis was performed because the small number of included studies (*n* = 4) did not meet the requirements for further refinement (Supplementary Figure 5.2).

#### Depression

BDI, SDS, HAMD were selected to evaluate depression in patients with PLP, and three RCTs (21, 22, 34, 37) with 142 patients were included. There was no statistical heterogeneity between studies by the X^2^ test (*p* = 0.06, I^2^ = 60%), and meta-analysis was performed. rTMS, TENS, and PNS had no effect on improving depression in PLP patients, with no statistically significant differences between groups [SMD = -0.44, 95% CI (−1.00, 0.12), *p* = 0.12] (Supplementary Figure 5.3).

### Neuromodulation effects on pain

We conduct subgroup analysis based on treatment types. Pain relief was evaluated using VAS as the outcome index. We found significant pooled effects for VAS reduction in rTMS [MD = -2.37, 95% CI (−4.35, −0.39), *p* = 0.02], tDCS [MD = -1.56, 95% CI (−2.37, −0.75), *p* = 0.0002], PNS [MD = -1.88, 95% CI (−3.05, −0.71), *p* = 0.002], central [MD = -0.55, 95% CI (−0.81, −0.30), *p* < 0.0001], and peripheral [SMD = -0.38, 95% CI (−0.73, −0.03), *p* = 0.03]. A subgroup analysis based on the type of control group showed that TENS studies [SMD = -0.46, 95% CI (−0.87, −0.06), *p* = 0.02], using sham stimulation as the control group, demonstrated a significant pooled effect on pain relief (Supplementary Table 1).

### Efficacy of rTMS with different treatment conditions in PLP patients

Subgroup analysis of rTMS was performed based on (1) Excitatory(≥5 Hz) and Inhibitory(≤1 Hz); (2) brain stimulation location; (3) treatment duration of >20 min and ≤20 min; (4) treatment period of >7 days and ≤ 7 days. Regarding VAS as the primary indicator, we found significant pain reduction at excitatory M1 (≥1 Hz) with duration ≤20 min [I^2^ = 0%, MD = -1.45, 95%CI (−2.78, −0.11), *p* = 0.03]. Only one study was in the subgroup of inhibitory DLPFC (≤1 Hz) with duration >20 min [MD = -4.48, 95%CI (−6.69, −2.27), *p* < 0.0001] and the pooled effect size cannot be calculated. We also found a significant effect size of pain reduction after >7 days treatment [MD = −4.35, 95%CI (−6.34, −2.36), *p* < 0.0001] (Supplementary Table 3).

### Efficacy of tDCS with different treatment conditions in PLP patients

Considering tDCS only. Pooled effects were analyzed based on the treatment types, intensity, duration, and period: (1) stimulation location and procedure; (2) treatment intensity of >1.5 mA and ≤1.5 mA; (3) duration of >15 min and ≤15 min; (4) treatment period of >1 week and ≤1 week. VAS was used as an outcome indicator to evaluate the improvement in pain relief. We found anodal M1 tDCS significantly reduced pain in PLP patients [I^2^ = 4%, MD = -1.60, 95%CI (−2.45, 0.74), *p* = 0.0003]. We also found tDCS with duration >15 min a significant pooled effect[I^2^ = 12%, MD = −1.91, 95%CI (−3.10, 0.72), *p* = 0.002]. No adverse effects are repoted to tDCS (Supplementary Table 2).

### Sensitivity analysis

Studies from the VAS and central subgroups were included in the sensitivity analysis. Excluding any single study, the combined results of the remaining studies remained statistically significant, consistent with the original combined results, indicating stable results. Other subgroups with fewer than ten studies were not included because they did not satisfy the essential elements for sensitivity analysis (Supplementary material 6.1–6.6).

## Discussion

Maladaptive plasticity versus persistent functional representation acknowledged that reorganization in the primary somatosensory cortex is not sufficient to explain phantom limb pain. Predictive coding framework (38) derived a three-step theory of the emergence and maintenance of PLP. When expectations differ from perceptual input, a prediction error occurs and evoke pains. Sensorimotor system increases salience processing and facilitate peripheral and central disinhibition to solve the prediction error, which lead to persistent pain. Neuromodulation or mirror therapy (39) can reduce predictive error and regulate nerual plasticity to restore sensorimotor system dysfunction.

The seventeen studies included in this review examined the effects of various neuromodulation techniques (six tDCS studies, four rTMS studies, four TENS studies, two PNS studies, and one NMES study) on PLP patients and showed an inhibitory effect on pain. However, the efficacy varied by treatment duration, frequency, and target.

Effects of central nervous system techniques on pain in patients with PLP tDCS and rTMS can modulate the excitatory-inhibitory balance of brain networks, acting on multiple stages of the predictive coding process: they can reduce aberrant sensory input and sensorimotor mismatch at the source, and also suppress the excessive amplification of pain signals downstream. Ultimately, these interventions work together to reduce the intensity and salience of the unresolved prediction error, breaking the vicious cycle of PLP and achieving pain relief.

Past research has predominantly focused on the neocortex, often overlooking the role of subcortical structures such as the cerebellum. Streng ML et al. (40) systematically explores the functional connections between the cerebellum and the mirror neuron system (MNS), particularly highlighting the cerebellum’s role in action observation (AO) and motor imagery (MI), as well as its implications for neuromodulation and rehabilitation. rTMS/tDCS may modulate Purkinje cell simple spike activity to improve predictive accuracy and reduce mismatches between expected and actual sensory feedback (41).

Our review comprised ten studies on central nervous system neuromodulation techniques, including tDCS (*n* = 6) and rTMS (*n* = 4). Six studies (25–30) on tDCS noted benefits in reducing PLP. Five studies (25–28, 30) targeted anodal M1 showed a significant reduction in PLP. Three study, respectively, targeted anodal PPC (25), anodal cerebellum (29) and cathoal PPC (25) showed no significant difference between intervention and control groups. Cathodal PPC of tDCS (25) reported a decrease of nonpainful phantom sensations. Due to the small number of included studies, we cannot suggest a potential dose response of this intervention.

Our exploratory subgroup analysis of rTMS included four RCTs (21–24) demonstrated pooled effects of excitatory/inhibitory and different brain stimulation sites. VAS scores declined with excitatory M1 rTMS. One study (24) used inhibitoy DLPFC reported an significant reduction of pain. Ahmed et al. believed that pain relief was related to increased serum beta-endorphin levels. Malavera et al. found that pain relief decreased after 30 days of treatment during follow-up, revealing the influence of time on the therapeutic effect of rTMS. We found that rTMS had significant effects when applied with high frequency (>1 Hz). This may be because rTMS at >1 Hz has an excitatory effect on the cerebral motor area and increases the content of brain-derived neurotrophic factor. Significant effects were observed at periods ≥7 days, while a period <7 days showed no significant effect. The pooled effect sizes of intensity below 1 Hz, duration >20 min could not be obtained due to insufficient studies.

rTMS (42) uses electromagnetic induction to cause more robust, direct neuronal depolarization and has broader effects on cortical excitability, neurochemistry and functional connectivity. tDCS uses weak electrical currents to subtly modulate membrane potentials. Its effects are more polarity-dependent and likely involve NMDA receptor-dependent synaptic plasticity. The effects of rTMS are longer-lasting in compare with tDCS, while tDCS lead a superiority pain alleviation which may related to the anti-inflamatory mechanism (43). Recent research indicates tDCS offers practicality for home-use but may require more sessions for sustained benefit (44).

### Effects of peripheral nervous system techniques on pain in patients with PLP

Peripheral disinhibition (reduced inhibitory control) allows excessive nociceptive input from sources like neuromas, ectopic nerve firing, or spinal ganglion changes to reach the brain. This input strengthens the cycle of PLP by fueling central disinhibition and prediction errors. Peripheral nerve regulation technologies directly reduce this abnormal input (38). Some patients with PLP also experience stump pain. After relief of stump pain, PLP will also be alleviated. Animal studies have shown that neuromas at the extremities of amputations release chemicals and enzymes, increasing the frequency of painful afferent impulses and the brain’s sensitivity to pain (45). The sensory input of stump abnormalities can induce sensorimotor cortex remodeling, although its effect on PLP is not yet clear (22, 46). This peripheral input may affect cortical remodeling after amputation (47, 48).

Our review comprised seven studies on peripheral nervous system neuromodulation techniques, including TENS (*n* = 4), PNS (*n* = 2), and NMES (*n* = 1). Meta-analysis showed that peripheral nerve regulation could relieve PLP based on VAS and MPQ. Subgroup analysis was performed based on different control groups. In the condition of sham stimulation as the control group, TENS had a positive impact on PLP based on VAS. Two PNS RCTs were selected, and the results showed that PNS significantly reduced pain intensity in PLP patients. However, it is premature to conclude the clinical efficacy of PNS in PLP patients, as the number of studies included in the PNS analysis was limited.

### Effects of neuromodulation techniques on depression in PLP patients

According to the Predictive Coding framework (38), the brain is an inference machine that continuously generates predictions and compares them with sensory inputs to minimize prediction errors (discrepancies between expectation and reality). Depression can be understood as a chronic, unresolved state of “interoceptive prediction error.” Neuroinflammation (49, 50), by impacting key neurotransmitter systems (HPA axis), neurogenesis, potently exacerbates and sustains this erroneous state (51). Regarding the studies included in this review, there is currently insufficient evidence to determine a definitive effect of neuromodulation treatments on depression. Three of the included studies indicated no significant effect, while Ahmed et al. (21) found that rTMS could reduce the anxiety and depression of PLP patients based on the Hamilton Scale. Ahmed et al. (21) and Malavera et al. (22) conduct excitatory M1 rTMS studies with frequency of 20 Hz and 10 Hz, respectively. Gilmore et al. (37) offered a minimally invasive option with potential long-term relief, possibly by modulating peripheral input and central plasticity. Kang (34) conduct a study of particularly intact-side TENS, provided a low-cost, non-invasive clinical approach emphasizing sensory reintegration and psychological factors in pain management. The four studies explored effective PLP treatments were heterogeneous across different levels (central, peripheral, integrated rehabilitation) and techniques (rTMS, PNS, TENS+OT). Whether neuromodulation techniques are an effective treatment for alleviating negative emotions in PLP patients still needs further evidence. Antonioni et al. (44) suggest that home-based tDCS could be a non-invasive, safe, and effective intervention for managing depression and patients with chronic pain, creating a precedent for its use in PLP.

## Limitations

A key limitation is the absence of subgroup analyses or meta-regressions to investigate the potential influence of important patient-level factors, such as the level and etiology of amputation, the presence of concomitant stump pain, and specific comorbidities. In routine clinical practice, response to treatment is likely heterogeneous and modulated by these variables. Consequently, the aggregate findings presented here should be extrapolated with caution to individual patients, as the extent to which they are applicable across these diverse clinical characteristics remains uncertain. Other limitation is the small sample size of 510 patients across the seventeen RCTs. The number of original studies on TENS, PNS, and NMES was too small to conduct a subgroup analysis based on different treatment intensities, durations, and periods. These factors contributed to the lack of diversity in the study results.

## Conclusion

This study found that rTMS and tDCS more effectively relieve PLP than other neuromodulation techniques. The analgesic effects of excitatory M1 rTMS [MD = −1.45, 95%CI (−2.78, −0.11), *p* = 0.03] and anodal M1 tDCS [MD = −1.60, 95% CI (−2.45, −0.74), *p* = 0.0002] exceed the minimal clinically important difference (MCID) of 1.4 for the VAS (52). However, these neuromodulation techniques do not appear to improve symptoms of depression. There is a better efficacy in tDCS treatment period lasts for >1 week and rTMS duration>20 min, but we cannot suggest a potential dose response due to the small number of the studies. There is a lack of evidences on the efficacy of invasive neuromodulation techniques (DRG, SCS and DBS, et al.). More RCTs with larger sample sizes and longer follow-up periods are necessary to evaluate the effects of neuromodulation techniques on PLP in the future.

## Data Availability

The original contributions presented in the study are included in the article/Supplementary material, further inquiries can be directed to the corresponding author.
