# Supplementary material for: Effects of neuromodulation techniques on pain and depression in patients with phantom limb pain: a systematic review and meta-analysis
Source: Front Neurol. 2025 Oct 22;16:1682650. doi: 10.3389/fneur.2025.1682650 (PMC12588162; doi:10.3389/fneur.2025.1682650)
Supplement: Supplementary file 1 [file Data_Sheet_1.pdf]

Supplementary materials：

Supplementary material 1: Search strategy

Database	Date	Terms
**MEDLINE PubMed**	**3/31/ 2025**	rTMS：<br>("Phantom Limb"[MESH] OR "Phantom"[tiab] OR "Phantom Limbs"[tiab] OR "Phantom Sensation"[tiab] OR "Phantom Sensations"[tiab] OR "Sensation"[tiab] OR "Sensations"[tiab] OR "Phantom Limb"[MeSH Terms] OR "Phantom Limb"[tiab] OR "Phantom Limb"[tiab] OR "Phantom Limb Pain"[tiab] OR "Phantom Limb Pains"[tiab] OR "Phantom Pain"[tiab] OR "Phantom Pains"[tiab] OR "stump pain"[tiab] OR "amputee*"[tiab] OR "amputation"[tiab] OR "Amputation Stump"[tiab]) AND ("Transcranial Magnetic Stimulation"[Mesh]) OR (Magnetic Stimulations, Transcranial) OR (Magnetic Stimulation, Transcranial) OR (Stimulations, Transcranial Magnetic) OR (Stimulation, Transcranial Magnetic) OR (Transcranial Magnetic Stimulations) OR (Transcranial Magnetic Stimulation, Paired Pulse) OR (Transcranial Magnetic Stimulation, Repetitive) OR (Transcranial Magnetic Stimulation, Single Pulse) AND ("randomized controlled trial"[All Fields] OR "randomized"[All Fields] OR "placebo"[All Fields])<br><br>tDCS：<br>("Phantom Limb"[MESH] OR "Phantom"[tiab] OR "Phantom Limbs"[tiab] OR "Phantom Sensation"[tiab] OR "Phantom Sensations"[tiab] OR "Sensation"[tiab] OR "Sensations"[tiab] OR "Phantom Limb"[MeSH Terms] OR "Phantom Limb"[tiab] OR "Phantom Limb"[tiab] OR "Phantom Limb Pain"[tiab] OR "Phantom Limb Pains"[tiab] OR "Phantom Pain"[tiab] OR "Phantom Pains"[tiab] OR "stump pain"[tiab] OR "amputee*"[tiab] OR "amputation"[tiab] OR "Amputation Stump"[tiab]) AND ("Transcranial Direct Current Stimulation"[All Fields] OR "tDCS"[All Fields] OR ("Transcranial Direct Current Stimulation"[MeSH Terms] OR ("transcranial"[All Fields] AND "direct"[All Fields] AND "current"[All Fields] AND "stimulation"[All Fields]) OR "Transcranial Direct Current Stimulation"[All Fields] OR ("cathodal"[All Fields] AND "stimulation"[All Fields] AND "transcranial"[All Fields] AND "direct"[All Fields] AND "current"[All Fields])) OR "Cathodal Stimulation tDCS"[All Fields] OR ("Transcranial Direct Current Stimulation"[MeSH Terms] OR ("transcranial"[All Fields] AND "direct"[All Fields] AND "current"[All Fields] AND "stimulation"[All Fields]) OR "Transcranial Direct Current Stimulation"[All Fields] OR ("cathodal"[All Fields] AND "stimulation"[All Fields] AND "tdcss"[All Fields])) OR "Stimulation tDCS Cathodal"[All Fields] OR (("stimulate"[All Fields] OR "stimulated"[All Fields] OR "stimulates"[All Fields] OR "stimulating"[All Fields] OR "stimulation"[All Fields] OR "stimulations"[All Fields] OR "stimulative"[All Fields] OR "stimulator"[All Fields] OR "stimulator s"[All Fields] OR "stimulators"[All Fields]) AND "tdcss"[All Fields]) OR "tDCS Cathodal Stimulation"[All Fields] OR ("tdcss"[All Fields] AND "cathodal"[All Fields] AND ("stimulate"[All Fields] OR "stimulated"[All Fields] OR "stimulates"[All Fields] OR "stimulating"[All Fields] OR "stimulation"[All Fields] OR

		"stimulations"[All Fields] OR "stimulative"[All Fields] OR "stimulator"[All Fields] OR "stimulator s"[All Fields] OR "stimulators"[All Fields])) OR "Transcranial Random Noise Stimulation"[All Fields] OR "Transcranial Alternating Current Stimulation"[All Fields] OR "Transcranial Electrical Stimulation"[All Fields] OR (("electric stimulation"[MeSH Terms] OR ("electric"[All Fields] AND "stimulation"[All Fields]) OR "electric stimulation"[All Fields] OR ("electrical"[All Fields] AND "stimulation"[All Fields]) OR "electrical stimulation"[All Fields]) AND "transcrania"[All Fields]) OR (("electric stimulation"[MeSH Terms] OR ("electric"[All Fields] AND "stimulation"[All Fields]) OR "electric stimulation"[All Fields] OR ("electrical"[All Fields] AND "stimulations"[All Fields]) OR "electrical stimulations"[All Fields]) AND ("transcranial"[All Fields] OR "transcranially"[All Fields])) OR "Stimulation Transcranial Electrical"[All Fields] OR (("stimulate"[All Fields] OR "stimulated"[All Fields] OR "stimulates"[All Fields] OR "stimulating"[All Fields] OR "stimulation"[All Fields] OR "stimulations"[All Fields] OR "stimulative"[All Fields] OR "stimulator"[All Fields] OR "stimulator s"[All Fields] OR "stimulators"[All Fields]) AND ("transcranial"[All Fields] OR "transcranially"[All Fields]) AND ("electricity"[MeSH Terms] OR "electricity"[All Fields] OR "electric"[All Fields] OR "electrical"[All Fields] OR "electrically"[All Fields] OR "electrics"[All Fields])) OR "Transcranial Electrical Stimulations"[All Fields] OR "Anodal Stimulation Transcranial Direct Current Stimulation"[All Fields] OR "Anodal Stimulation tDCS"[All Fields] OR ("Transcranial Direct Current Stimulation"[MeSH Terms] OR ("transcranial"[All Fields] AND "direct"[All Fields] AND "current"[All Fields] AND "stimulation"[All Fields]) OR "Transcranial Direct Current Stimulation"[All Fields] OR ("anodal"[All Fields] AND "stimulation"[All Fields] AND "tdcss"[All Fields])) OR "Stimulation tDCS Anodal"[All Fields] OR (("stimulate"[All Fields] OR "stimulated"[All Fields] OR "stimulates"[All Fields] OR "stimulating"[All Fields] OR "stimulation"[All Fields] OR "stimulations"[All Fields] OR "stimulative"[All Fields] OR "stimulator"[All Fields] OR "stimulator s"[All Fields] OR "stimulators"[All Fields]) AND "tdcss"[All Fields] AND "anodal"[All Fields]) OR "tDCS Anodal Stimulation"[All Fields] OR ("tdcss"[All Fields] AND "anodal"[All Fields] AND ("stimulate"[All Fields] OR "stimulated"[All Fields] OR "stimulates"[All Fields] OR "stimulating"[All Fields] OR "stimulation"[All Fields] OR "stimulations"[All Fields] OR "stimulative"[All Fields] OR "stimulator"[All Fields] OR "stimulator s"[All Fields] OR "stimulators"[All Fields])) OR "Repetitive Transcranial Electrical Stimulation"[All Fields]) AND ("randomized controlled trial"[All Fields] OR "randomized"[All Fields] OR "placebo"[All Fields])<br><br>TENS：<br>("Phantom Limb"[MESH] OR "Phantom"[tiab] OR "Phantom Limbs"[tiab] OR "Phantom Sensation"[tiab] OR "Phantom Sensations"[tiab] OR "Sensation"[tiab] OR "Sensations"[tiab] OR "Phantom Limb"[MeSH Terms] OR "Phantom Limb"[tiab] OR "Phantom Limb"[tiab] OR "Phantom Limb Pain"[tiab] OR "Phantom Limb Pains"[tiab] OR "Phantom Pain"[tiab] OR "Phantom Pains"[tiab] OR "stump pain"[tiab] OR "amputee*"[tiab] OR "amputation"[tiab] OR "Amputation Stump"[tiab]) AND

		("Transcutaneous Electric Nerve Stimulation"[All Fields] OR "Electric Stimulation Transcutaneous"[All Fields] OR "Stimulation Transcutaneous Electric"[All Fields] OR "Transcutaneous Electric Stimulation"[All Fields] OR "Percutaneous Electric Nerve Stimulation"[All Fields] OR "Electrical Stimulation Transcutaneous"[All Fields] OR "Transcutaneous Electrical Stimulation"[All Fields] OR "Transdermal Electrostimulation"[All Fields] OR (("electrostimulated"[All Fields] OR "electrostimulating"[All Fields] OR "electrostimulation"[All Fields] OR "electrostimulations"[All Fields] OR "electrostimulator"[All Fields] OR "electrostimulators"[All Fields]) AND ("administration, cutaneous"[MeSH Terms] OR ("administration"[All Fields] AND "cutaneous"[All Fields]) OR "cutaneous administration"[All Fields] OR "transdermal"[All Fields] OR "transdermally"[All Fields] OR "transdermals"[All Fields] OR "transdermic"[All Fields] OR "transdermically"[All Fields])) OR "Percutaneous Electrical Nerve Stimulation"[All Fields] OR "Transcutaneous Electrical Nerve Stimulation"[All Fields] OR "Transcutaneous Nerve Stimulation"[All Fields] OR "Nerve Stimulation Transcutaneous"[All Fields] OR "Stimulation Transcutaneous Nerve"[All Fields] OR "TENS"[All Fields] OR "Percutaneous Neuromodulation Therapy"[All Fields] OR (("neuromodulate"[All Fields] OR "neuromodulating"[All Fields] OR "neuromodulation"[All Fields] OR "neuromodulations"[All Fields] OR "neuromodulative"[All Fields] OR "neurotransmitter agents"[Pharmacological Action] OR "neurotransmitter agents"[MeSH Terms] OR ("neurotransmitter"[All Fields] AND "agents"[All Fields]) OR "neurotransmitter agents"[All Fields] OR "neuromodulator"[All Fields] OR "neuromodulators"[All Fields]) AND ("therapeutics"[MeSH Terms] OR "therapeutics"[All Fields] OR "therapies"[All Fields] OR "therapy"[MeSH Subheading] OR "therapy"[All Fields] OR "therapy s"[All Fields] OR "therapys"[All Fields]) AND ("percutaneous"[All Fields] OR "percutaneously"[All Fields] OR "percutanous"[All Fields])) OR "Percutaneous Neuromodulation Therapies"[All Fields] OR (("therapeutics"[MeSH Terms] OR "therapeutics"[All Fields] OR "therapies"[All Fields] OR "therapy"[MeSH Subheading] OR "therapy"[All Fields] OR "therapy s"[All Fields] OR "therapys"[All Fields]) AND ("percutaneous"[All Fields] OR "percutaneously"[All Fields] OR "percutanous"[All Fields]) AND ("neuromodulate"[All Fields] OR "neuromodulating"[All Fields] OR "neuromodulation"[All Fields] OR "neuromodulations"[All Fields] OR "neuromodulative"[All Fields] OR "neurotransmitter agents"[Pharmacological Action] OR "neurotransmitter agents"[MeSH Terms] OR ("neurotransmitter"[All Fields] AND "agents"[All Fields]) OR "neurotransmitter agents"[All Fields] OR "neuromodulator"[All Fields] OR "neuromodulators"[All Fields])) OR "Percutaneous Electrical Neuromodulation"[All Fields] OR (("electricity"[MeSH Terms] OR "electricity"[All Fields] OR "electric"[All Fields] OR "electrical"[All Fields] OR "electrically"[All Fields] OR "electrics"[All Fields]) AND ("neuromodulate"[All Fields] OR "neuromodulating"[All Fields] OR "neuromodulation"[All Fields] OR "neuromodulations"[All Fields] OR "neuromodulative"[All Fields] OR "neurotransmitter agents"[Pharmacological Action] OR "neurotransmitter agents"[MeSH Terms] OR ("neurotransmitter"[All Fields] AND "agents"[All Fields])

		OR "neurotransmitter agents"[All Fields] OR "neuromodulator"[All Fields] OR "neuromodulators"[All Fields]) AND ("percutaneous"[All Fields] OR "percutaneously"[All Fields] OR "percutanous"[All Fields])) OR (("electricity"[MeSH Terms] OR "electricity"[All Fields] OR "electric"[All Fields] OR "electrical"[All Fields] OR "electrically"[All Fields] OR "electrics"[All Fields]) AND ("neuromodulate"[All Fields] OR "neuromodulating"[All Fields] OR "neuromodulation"[All Fields] OR "neuromodulations"[All Fields] OR "neuromodulative"[All Fields] OR "neurotransmitter agents"[Pharmacological Action] OR "neurotransmitter agents"[MeSH Terms] OR ("neurotransmitter"[All Fields] AND "agents"[All Fields]) OR "neurotransmitter agents"[All Fields] OR "neuromodulator"[All Fields] OR "neuromodulators"[All Fields]) AND ("percutaneous"[All Fields] OR "percutaneously"[All Fields] OR "percutanous"[All Fields])) OR (("neuromodulate"[All Fields] OR "neuromodulating"[All Fields] OR "neuromodulation"[All Fields] OR "neuromodulations"[All Fields] OR "neuromodulative"[All Fields] OR "neurotransmitter agents"[Pharmacological Action] OR "neurotransmitter agents"[MeSH Terms] OR ("neurotransmitter"[All Fields] AND "agents"[All Fields]) OR "neurotransmitter agents"[All Fields] OR "neuromodulator"[All Fields] OR "neuromodulators"[All Fields]) AND ("percutaneous"[All Fields] OR "percutaneously"[All Fields] OR "percutanous"[All Fields]) AND ("electricity"[MeSH Terms] OR "electricity"[All Fields] OR "electric"[All Fields] OR "electrical"[All Fields] OR "electrically"[All Fields] OR "electrics"[All Fields])) OR (("neuromodulate"[All Fields] OR "neuromodulating"[All Fields] OR "neuromodulation"[All Fields] OR "neuromodulations"[All Fields] OR "neuromodulative"[All Fields] OR "neurotransmitter agents"[Pharmacological Action] OR "neurotransmitter agents"[MeSH Terms] OR ("neurotransmitter"[All Fields] AND "agents"[All Fields]) OR "neurotransmitter agents"[All Fields] OR "neuromodulator"[All Fields] OR "neuromodulators"[All Fields]) AND ("percutaneous"[All Fields] OR "percutaneously"[All Fields] OR "percutanous"[All Fields]) AND ("electricity"[MeSH Terms] OR "electricity"[All Fields] OR "electric"[All Fields] OR "electrical"[All Fields] OR "electrically"[All Fields] OR "electrics"[All Fields])) OR ("Transcutaneous Electric Nerve Stimulation"[MeSH Terms] OR ("transcutaneous"[All Fields] AND "electric"[All Fields] AND "nerve"[All Fields] AND "stimulation"[All Fields]) OR "Transcutaneous Electric Nerve Stimulation"[All Fields] OR ("percutaneous"[All Fields] AND "electrical"[All Fields] AND "neuromodulations"[All Fields])) OR "Analgesic Cutaneous Electrostimulation"[All Fields] OR (("cutaneous"[All Fields] OR "cutaneously"[All Fields] OR "cutanous"[All Fields]) AND ("electrostimulated"[All Fields] OR "electrostimulating"[All Fields] OR "electrostimulation"[All Fields] OR "electrostimulations"[All Fields] OR "electrostimulator"[All Fields] OR "electrostimulators"[All Fields]) AND ("analgesic s"[All Fields] OR "analgesically"[All Fields] OR "analgesics"[Pharmacological Action] OR "analgesics"[MeSH Terms] OR "analgesics"[All Fields] OR "analgesic"[All Fields])) OR (("electrostimulated"[All Fields] OR "electrostimulating"[All Fields] OR "electrostimulation"[All Fields] OR "electrostimulations"[All Fields] OR "electrostimulator"[All Fields] OR "electrostimulators"[All Fields]) AND ("analgesic s"[All Fields] OR "analgesically"[All

		Fields] OR "analgesics"[Pharmacological Action] OR "analgesics"[MeSH Terms] OR "analgesics"[All Fields] OR "analgesic"[All Fields]) AND ("cutaneous"[All Fields] OR "cutaneously"[All Fields] OR "cutanous"[All Fields])) OR ("Transcutaneous Electric Nerve Stimulation"[MeSH Terms] OR ("transcutaneous"[All Fields] AND "electric"[All Fields] AND "nerve"[All Fields] AND "stimulation"[All Fields]) OR "Transcutaneous Electric Nerve Stimulation"[All Fields] OR "electroanalgesia"[All Fields] OR ("Transcutaneous Electric Nerve Stimulation"[MeSH Terms] OR ("transcutaneous"[All Fields] AND "electric"[All Fields] AND "nerve"[All Fields] AND "stimulation"[All Fields]) OR "Transcutaneous Electric Nerve Stimulation"[All Fields]))) AND ("randomized controlled trial"[All Fields] OR "randomized"[All Fields] OR "placebo"[All Fields])<br><br>NMES &PNS<br>("Phantom Limb"[MESH] OR "Phantom"[tiab] OR "Phantom Limbs"[tiab] OR "Phantom Sensation"[tiab] OR "Phantom Sensations"[tiab] OR "Sensation"[tiab] OR "Sensations"[tiab] OR "Phantom Limb"[MeSH Terms] OR "Phantom Limb"[tiab] OR "Phantom Limb"[tiab] OR "Phantom Limb Pain"[tiab] OR "Phantom Limb Pains"[tiab] OR "Phantom Pain"[tiab] OR "Phantom Pains"[tiab] OR "stump pain"[tiab] OR "amputee*"[tiab] OR "amputation"[tiab] OR "Amputation Stump"[tiab]) AND ("Neuromuscular Electrical Stimulation"[tiab] OR "Electrical Stimulation"[tiab] OR "Electrical Stimulation"[tiab] OR "Neuromuscular Stimulation"[tiab] OR "Electrical Neuromuscular Stimulation"[tiab] OR "Neuromuscular Electrical"[tiab] OR "NMES"[tiab] OR "peripheral nerve stimulation"[TIAB] OR "PNS"[TIAB] OR "peripheral somatosensory stimulation"[TIAB] OR "PSS"[TIAB] OR "nerve stimulation"[TIAB] or peripherical stimulation[TIAB]) AND ("randomized controlled trial"[All Fields] OR "randomized"[All Fields] OR "placebo"[All Fields])
**The Cochrane Library**	**3/31/ 2025**	rTMS<br>((Phantom Limb):ti,ab,kw OR (Limb):ti,ab,kw OR (Phantom):ti,ab,kw OR (Phantom Limbs):ti,ab,kw OR (Phantom Sensation):ti,ab,kw OR (Phantom Sensations):ti,ab,kw OR (Sensation):ti,ab,kw OR (Sensations):ti,ab,kw OR (Pseudomelia):ti,ab,kw OR (Pseudomelia):ti,ab,kw OR (Phantom Limb Pain):ti,ab,kw OR (Phantom Limb Pains):ti,ab,kw OR (Phantom Pain):ti,ab,kw OR (Phantom Pains):ti,ab,kw) AND ((Transcranial Magnetic Stimulation):ti,ab,kw OR (Magnetic Stimulation Transcranial):ti,ab,kw OR (Magnetic Stimulations Transcranial):ti,ab,kw OR (Stimulation Transcranial Magnetic):ti,ab,kw OR (Stimulations Transcranial Magnetic):ti,ab,kw OR (Transcranial Magnetic Stimulations):ti,ab,kw OR (Transcranial Magnetic Stimulation Single Pulse):ti,ab,kw OR (Transcranial Magnetic Stimulation Paired Pulse):ti,ab,kw OR (Transcranial Magnetic Stimulation Repetitive):ti,ab,kw) AND ((randomized controlled trial):ti,ab,kw OR (randomized):ti,ab,kw OR (placebo):ti,ab,kw)<br>tDCS<br>((Phantom Limb):ti,ab,kw OR (Limb):ti,ab,kw OR (Phantom):ti,ab,kw OR (Phantom Limbs):ti,ab,kw OR (Phantom Sensation):ti,ab,kw OR (Phantom Sensations):ti,ab,kw OR (Sensation):ti,ab,kw OR (Sensations):ti,ab,kw OR (Pseudomelia):ti,ab,kw OR

		(Pseudomelia):ti,ab,kw OR (Phantom Limb Pain):ti,ab,kw OR (Phantom Limb Pains):ti,ab,kw OR (Phantom Pain):ti,ab,kw OR (Phantom Pains):ti,ab,kw) AND ((Transcranial Direct Current Stimulation):ti,ab,kw OR (tDCS):ti,ab,kw OR (Cathodal Stimulation Transcranial Direct Current Stimulation):ti,ab,kw OR (Cathodal Stimulation tDCS):ti,ab,kw OR (Cathodal Stimulation tDCSs):ti,ab,kw OR (Stimulation tDCS Cathodal):ti,ab,kw OR (Stimulation tDCSs Cathodal):ti,ab,kw OR (tDCS Cathodal Stimulation):ti,ab,kw OR (tDCSs Cathodal Stimulation):ti,ab,kw OR (Transcranial Random Noise Stimulation):ti,ab,kw OR (Transcranial Alternating Current Stimulation):ti,ab,kw OR (Transcranial Electrical Stimulation):ti,ab,kw OR (Electrical Stimulation Transcranial):ti,ab,kw OR (Electrical Stimulations Transcranial):ti,ab,kw OR (Stimulation Transcranial Electrical):ti,ab,kw OR (Stimulations Transcranial Electrical):ti,ab,kw OR (Transcranial Electrical Stimulations):ti,ab,kw OR (Anodal Stimulation Transcranial Direct Current Stimulation):ti,ab,kw OR (Anodal Stimulation tDCS):ti,ab,kw OR (Anodal Stimulation tDCSs):ti,ab,kw OR (Stimulation tDCS Anodal):ti,ab,kw OR (Stimulation tDCSs Anodal):ti,ab,kw OR (tDCS Anodal Stimulation):ti,ab,kw OR (tDCSs Anodal Stimulation):ti,ab,kw OR (Repetitive Transcranial Electrical Stimulation):ti,ab,kw) AND ((randomized controlled trial):ti,ab,kw OR (randomized):ti,ab,kw OR (placebo):ti,ab,kw)<br>TENS<br>((Phantom Limb):ti,ab,kw OR (Limb):ti,ab,kw OR (Phantom):ti,ab,kw OR (Phantom Limbs):ti,ab,kw OR (Phantom Sensation):ti,ab,kw OR (Phantom Sensations):ti,ab,kw OR (Sensation):ti,ab,kw OR (Sensations):ti,ab,kw OR (Pseudomelia):ti,ab,kw OR (Pseudomelia):ti,ab,kw OR (Phantom Limb Pain):ti,ab,kw OR (Phantom Limb Pains):ti,ab,kw OR (Phantom Pain):ti,ab,kw OR (Phantom Pains):ti,ab,kw) AND ((Transcutaneous Electric Nerve Stimulation):ti,ab,kw OR (Electric Stimulation Transcutaneous):ti,ab,kw OR (Stimulation Transcutaneous Electric):ti,ab,kw OR (Transcutaneous Electric Stimulation):ti,ab,kw OR (Percutaneous Electric Nerve Stimulation):ti,ab,kw OR (Electrical Stimulation Transcutaneous):ti,ab,kw OR (Transcutaneous Electrical Stimulation):ti,ab,kw OR (Transdermal Electrostimulation):ti,ab,kw OR (Electrostimulation Transdermal):ti,ab,kw OR (Percutaneous Electrical Nerve Stimulation):ti,ab,kw OR (Transcutaneous Electrical Nerve Stimulation):ti,ab,kw OR (Transcutaneous Nerve Stimulation):ti,ab,kw OR (Nerve Stimulation Transcutaneous):ti,ab,kw OR (Stimulation Transcutaneous Nerve):ti,ab,kw OR (TENS):ti,ab,kw OR (Percutaneous Neuromodulation Therapy):ti,ab,kw OR (Neuromodulation Therapy Percutaneous):ti,ab,kw OR (Percutaneous Neuromodulation Therapies):ti,ab,kw OR (Therapy Percutaneous Neuromodulation):ti,ab,kw OR (Percutaneous Electrical Neuromodulation):ti,ab,kw OR (Electrical Neuromodulation Percutaneous):ti,ab,kw OR (Electrical Neuromodulations Percutaneous):ti,ab,kw OR (Neuromodulation Percutaneous Electrical):ti,ab,kw OR (Neuromodulations Percutaneous Electrical):ti,ab,kw OR (Percutaneous Electrical Neuromodulations):ti,ab,kw OR (Analgesic Cutaneous Electrostimulation):ti,ab,kw OR (Cutaneous Electrostimulation Analgesic):ti,ab,kw OR (Electrostimulation Analgesic Cutaneous):ti,ab,kw OR (Electroanalgesia OR Electroanalgesias):ti,ab,kw) AND

		((randomized controlled trial):ti,ab,kw OR (randomized):ti,ab,kw OR (placebo):ti,ab,kw)<br>NMES and PNS<br>((Phantom Limb):ti,ab,kw OR (Limb):ti,ab,kw OR (Phantom):ti,ab,kw OR (Phantom Limbs):ti,ab,kw OR (Phantom Sensation):ti,ab,kw OR (Phantom Sensations):ti,ab,kw OR (Sensation):ti,ab,kw OR (Sensations):ti,ab,kw OR (Pseudomelia):ti,ab,kw OR (Pseudomelia):ti,ab,kw OR (Phantom Limb Pain):ti,ab,kw OR (Phantom Limb Pains):ti,ab,kw OR (Phantom Pain):ti,ab,kw OR (Phantom Pains):ti,ab,kw) AND ((neuromuscular electrical stimulation):ti,ab,kw OR (neuromuscular electrical stimulation):ti,ab,kw OR (neuromuscular electric stimulation):ti,ab,kw OR (neuromuscular electricostimulation):ti,ab,kw OR (NMES):ti,ab,kw OR (PNS):ti,ab,kw OR (peripheral nerve stimulation):ti,ab,kw OR (peripheral nerve stimulation):ti,ab,kw OR (peripheral somatosensory stimulation):ti,ab,kw OR (PSS):ti,ab,kw OR (nerve stimulation):ti,ab,kw OR (peripherical stimulation):ti,ab,kw) AND ((randomized controlled trial):ti,ab,kw OR (randomized):ti,ab,kw OR (placebo):ti,ab,kw)
**EMBASE**	**3/31/ 2025**	rTMS<br>('phantom limb'/exp OR 'phantom limb sensation' OR 'phantom limb syndrome' OR 'phantom sensation' OR 'phantom limb' OR 'phantom' OR 'phantom sensations' OR 'pseudomelia' OR 'phantom limb pain' OR 'phantom limb pains' OR 'phantom pain' OR 'phantom pains') AND ('transcranial magnetic stimulation'/exp OR 'magnetic stimulation, transcranial' OR 'stimulation, transcranial magnetic' OR 'transcranial magnetic stimulation' OR 'magnetic stimulation transcranial' OR 'magnetic stimulations transcranial' OR 'stimulation transcranial magnetic' OR 'stimulations transcranial magnetic' OR 'transcranial magnetic stimulations' OR 'transcranial magnetic stimulation single pulse' OR 'transcranial magnetic stimulation paired pulse' OR 'transcranial magnetic stimulation repetitive') AND ('randomized controlled trial'/exp OR 'controlled trial, randomized' OR 'randomised controlled study' OR 'randomised controlled trial' OR 'randomized controlled study' OR 'trial, randomized controlled' OR 'randomized controlled trial')<br>tDCS<br>('phantom limb'/exp OR 'phantom limb sensation' OR 'phantom limb syndrome' OR 'phantom sensation' OR 'pseudomelia' OR 'phantom limb' OR 'phantom' OR 'phantom sensations' OR 'Pseudomelia' OR 'Phantom Limb Pain' OR 'Phantom Limb Pains' OR 'Phantom Pain' OR 'Phantom Pains') AND ('Transcranial Direct Current Stimulation'/exp OR 'tDCS' OR 'Cathodal Stimulation Transcranial Direct Current Stimulation' OR 'Cathodal Stimulation tDCS' OR 'Cathodal Stimulation tDCSs' OR 'Stimulation tDCS, Cathodal' OR 'Stimulation tDCSs, Cathodal' OR 'tDCS, Cathodal Stimulation' OR 'tDCSs, Cathodal Stimulation' OR 'Transcranial Random Noise Stimulation' OR 'Transcranial Alternating Current Stimulation' OR 'Transcranial Electrical Stimulation' OR 'Electrical Stimulation, Transcranial' OR 'Electrical Stimulations, Transcranial' OR 'Stimulation, Transcranial Electrical' OR 'Stimulations, Transcranial Electrical' OR 'Transcranial Electrical Stimulations' OR 'Anodal Stimulation Transcranial Direct Current Stimulation' OR 'Anodal Stimulation tDCS' OR 'Anodal Stimulation tDCSs' OR

		'Stimulation tDCS, Anodal' OR 'Stimulation tDCSs, Anodal' OR 'tDCS, Anodal Stimulation' OR 'tDCSs, Anodal Stimulation' OR 'Repetitive Transcranial Electrical Stimulation') AND ('randomized controlled trial'/exp OR 'controlled trial, randomized' OR 'randomised controlled study' OR 'randomised controlled trial' OR 'randomized controlled study' OR 'trial, randomized controlled' OR 'randomized controlled trial')<br>TENS<br>('phantom limb'/exp OR 'phantom limb sensation' OR 'phantom limb syndrome' OR 'phantom sensation' OR 'pseudomelia' OR 'phantom limb' OR 'phantom' OR 'phantom sensations' OR 'Pseudomelia' OR 'Phantom Limb Pain' OR 'Phantom Limb Pains' OR 'Phantom Pain' OR 'Phantom Pains') AND ('Transcutaneous Electric Nerve Stimulation'/exp OR 'Electric Stimulation, Transcutaneous' OR 'Stimulation, Transcutaneous Electric' OR 'Transcutaneous Electric Stimulation' OR 'Percutaneous Electric Nerve Stimulation' OR 'Electrical Stimulation, Transcutaneous' OR 'Transcutaneous Electrical Stimulation' OR 'Transdermal Electrostimulation' OR 'Electrostimulation, Transdermal' OR 'Percutaneous Electrical Nerve Stimulation' OR 'Transcutaneous Electrical Nerve Stimulation' OR 'Transcutaneous Nerve Stimulation' OR 'Nerve Stimulation, Transcutaneous' OR 'Stimulation, Transcutaneous Nerve' OR 'TENS' OR 'Percutaneous Neuromodulation Therapy' OR 'Neuromodulation Therapy, Percutaneous' OR 'Percutaneous Neuromodulation Therapies' OR 'Therapy, Percutaneous Neuromodulation' OR 'Percutaneous Electrical Neuromodulation' OR 'Electrical Neuromodulation, Percutaneous' OR 'Electrical Neuromodulations, Percutaneous' OR 'Neuromodulation, Percutaneous Electrical' OR 'Neuromodulations, Percutaneous Electrical' OR 'Percutaneous Electrical Neuromodulations' OR 'Analgesic Cutaneous Electrostimulation' OR 'Cutaneous Electrostimulation, Analgesic' OR 'Electrostimulation, Analgesic Cutaneous' OR 'Electroanalgesia' OR 'Electroanalgesias') AND ('randomized controlled trial'/exp OR 'controlled trial, randomized' OR 'randomised controlled study' OR 'randomised controlled trial' OR 'randomized controlled study' OR 'trial, randomized controlled' OR 'randomized controlled trial')<br>NMES and PNS<br>('Phantom Limb' OR 'Phantom' OR 'Phantom Limbs' OR 'Phantom Sensation' OR 'Phantom Sensations' OR 'Sensation' OR 'Sensations' OR 'Pseudomelia' OR 'Pseudomelia' OR 'Phantom Limb Pain' OR 'Phantom Limb Pains' OR 'Phantom Pain' OR 'Phantom Pains') AND ('neuromuscular electrical stimulation' OR 'neuromuscular electrical stimulation' OR 'neuromuscular electric stimulation' OR 'neuromuscular electricostimulation' OR 'NMES' OR 'PNS' OR 'peripheral nerve stimulation' OR 'peripheral nerve stimulation' OR 'peripheral somatosensory stimulation' OR 'PSS' OR 'nerve stimulation' OR 'peripherical stimulation') AND ('randomized controlled trial' OR 'randomized' OR 'placebo')

**Web of Science**	**3/31/ 2025**	rTMS<br>(TS=(Phantom Limb OR Phantom OR Phantom Limbs OR Phantom Sensation OR Phantom Sensations OR Pseudomelia OR Pseudomelia OR Phantom Limb Pain OR Phantom Limb Pains OR Phantom Pain OR Phantom Painsimb OR Phantom OR Phantom Limbs OR Phantom Sensation OR Phantom Sensations OR pseudovelia OR pseudovelia OR Phantom Limb Pain OR Phantom Limb Pains OR Phantom Pain OR Phantom Pains)) AND(TS=(Transcranial Magnetic Stimulation OR Magnetic Stimulation Transcranial OR Magnetic Stimulations Transcranial OR Stimulation Transcranial Magnetic OR Stimulations Transcranial Magnetic OR Transcranial Magnetic Stimulations OR Transcranial Magnetic Stimulation Single Pulse OR Transcranial Magnetic Stimulation Paired Pulse OR Transcranial Magnetic Stimulation Repetitive)) AND (TS=(randomized controlled trial OR randomized OR placebo))<br>tDCS<br>(TS=(Phantom Limb OR Phantom OR Phantom Limbs OR Phantom Sensation OR Phantom Sensations OR Pseudomelia OR Pseudomelia OR Phantom Limb Pain OR Phantom Limb Pains OR Phantom Pain OR Phantom Painsimb OR Phantom OR Phantom Limbs OR Phantom Sensation OR Phantom Sensations OR pseudovelia OR pseudovelia OR Phantom Limb Pain OR Phantom Limb Pains OR Phantom Pain OR Phantom Pains)) AND(TS=(Transcranial Direct Current Stimulation OR tDCS OR Cathodal Stimulation Transcranial Direct Current Stimulation OR Cathodal Stimulation tDCS OR Cathodal Stimulation tDCSs OR Stimulation tDCS Cathodal OR Stimulation tDCSs Cathodal OR tDCS Cathodal Stimulation OR tDCSs Cathodal Stimulation OR Transcranial Random Noise Stimulation OR Transcranial Alternating Current Stimulation OR Transcranial Electrical Stimulation OR Electrical Stimulation Transcranial OR Electrical Stimulations Transcranial OR Stimulation Transcranial Electrical OR Stimulations Transcranial Electrical OR Transcranial Electrical Stimulations OR Anodal Stimulation Transcranial Direct Current Stimulation OR Anodal Stimulation tDCS OR Anodal Stimulation tDCSs OR Stimulation tDCS Anodal OR Stimulation tDCSs Anodal OR tDCS Anodal Stimulation OR tDCSs Anodal Stimulation OR Repetitive Transcranial Electrical Stimulation)) AND (TS=(randomized controlled trial OR randomized OR placebo))<br>TENS<br>(TS=(Phantom Limb OR Phantom OR Phantom Limbs OR Phantom Sensation OR Phantom Sensations OR Pseudomelia OR Pseudomelia OR Phantom Limb Pain OR Phantom Limb Pains OR Phantom Pain OR Phantom Painsimb OR Phantom OR Phantom Limbs OR Phantom Sensation OR Phantom Sensations OR pseudovelia OR pseudovelia OR Phantom Limb Pain OR Phantom Limb Pains OR Phantom Pain OR Phantom Pains)) AND(TS=(Transcutaneous Electric Nerve Stimulation OR Electric Stimulation Transcutaneous OR Stimulation Transcutaneous Electric OR Transcutaneous Electric Stimulation OR Percutaneous Electric Nerve Stimulation OR Electrical Stimulation Transcutaneous OR Transcutaneous Electrical Stimulation OR Transdermal Electrostimulation OR Electrostimulation Transdermal OR Percutaneous Electrical Nerve Stimulation OR Transcutaneous Electrical Nerve

		Stimulation OR Transcutaneous Nerve Stimulation OR Nerve Stimulation Transcutaneous OR Stimulation Transcutaneous Nerve OR TENS OR Percutaneous Neuromodulation Therapy OR Neuromodulation Therapy Percutaneous OR Percutaneous Neuromodulation Therapies OR Therapy Percutaneous Neuromodulation OR Percutaneous Electrical Neuromodulation OR Electrical Neuromodulation Percutaneous OR Electrical Neuromodulations Percutaneous OR Neuromodulation Percutaneous Electrical OR Neuromodulations Percutaneous Electrical OR Percutaneous Electrical Neuromodulations OR Analgesic Cutaneous Electrostimulation OR Cutaneous Electrostimulation Analgesic OR Electrostimulation Analgesic Cutaneous OR Electroanalgesia OR Electroanalgesias)) AND (TS=(randomized controlled trial OR randomized OR placebo))<br>NMES&PNS<br>(TS=(Phantom Limb OR Phantom OR Phantom Limbs OR Phantom Sensation OR Phantom Sensations OR Pseudomelia OR Pseudomelia OR Phantom Limb Pain OR Phantom Limb Pains OR Phantom Pain OR Phantom Painsimb OR Phantom OR Phantom Limbs OR Phantom Sensation OR Phantom Sensations OR pseudovelia OR pseudovelia OR Phantom Limb Pain OR Phantom Limb Pains OR Phantom Pain OR Phantom Pains)) AND(TS=(neuromuscular electrical stimulation OR neuromuscular electric stimulation OR neuromuscular electric stimulation OR neuromuscular electricostimulation OR NMES OR PNS OR peripheral nerve stimulation OR peripheral nerve stimulation OR peripheral somatosensory stimulation OR PSS OR nerve stimulation OR peripherical stimulation)) AND (TS=(randomized controlled trial OR randomized OR placebo))
**Scopus**	**3/31/ 2025**	rTMS<br><br>(TITLE-ABS-KEY ("Phantom Limb" OR "Phantom" OR "Phantom Limbs" OR "Phantom Sensation" OR "Phantom Sensations" OR "Sensation" OR "Sensations" OR "Pseudomelia" OR "Pseudomelia " OR "Phantom Limb Pain" OR "Phantom Limb Pains" OR "Phantom Pain" OR "Phantom Pains")) AND (TITLE-ABS-KEY ("Transcranial Magnetic Stimulation" OR "Magnetic Stimulation Transcranial" OR "Magnetic Stimulations Transcranial" OR "Stimulation Transcranial Magnetic" OR "Stimulations Transcranial Magnetic" OR "Transcranial Magnetic Stimulations" OR "Transcranial Magnetic Stimulation Single Pulse" OR "Transcranial Magnetic Stimulation Paired Pulse" OR "Transcranial Magnetic Stimulation Repetitive")) AND (TITLE-ABS-KEY ("randomized controlled trial" OR "randomized" OR "placebo"))<br><br>tDCS<br><br>(TITLE-ABS-KEY ("Phantom Limb" OR "Phantom" OR "Phantom Limbs" OR "Phantom Sensation" OR "Phantom Sensations" OR "Sensation" OR "Sensations" OR "Pseudomelia" OR "Pseudomelia" O R "Phantom Limb Pain" OR "Phantom Limb Pains" OR "Phantom Pain" OR "Phantom

		Pains")) AND TITLE-ABS-KEY (("Transcranial Direct Current Stimulation" OR "tDCS" OR "Cathodal Stimulation Transcranial Direct Current Stimulation" OR "Cathodal Stimulation tDCS" OR "Cathodal Stimulation tDCSs" OR "Stimulation tDCS Cathodal" OR "Stimulation tDCSs Cathoda" OR "tDCS Cathodal Stimulation" OR "tDCSs Cathodal Stimulation" OR "Transcranial Random Noise Stimulation" OR "Transcranial Alternating Current Stimulation" OR "Transcranial Electrical Stimulation" OR "Electrical Stimulation Transcrania" OR "Electrical Stimulations Transcranial" OR "Stimulation Transcranial Electrical" OR "Stimulations Transcranial Electrical" OR "Transcranial Electrical Stimulations" OR "Anodal Stimulation Transcranial Direct Current Stimulation" OR "Anodal Stimulation tDCS" OR "Anodal Stimulation tDCSs" OR "Stimulation tDCS Anodal" OR "Stimulation tDCSs Anodal" OR "tDCS Anodal Stimulation" OR "tDCSs Anodal Stimulation" OR "Repetitive Transcranial Electrical Stimulation")) AND (TITLE-ABS-KEY ("randomized controlled trial" OR "randomized" OR "placebo"))<br><br>TENS,NMES and PNS<br><br>(TITLE-ABS-KEY (“Phantom Limb” OR “Phantom” OR “Phantom Limbs” OR “Phantom Sensation” OR “Phantom Sensations” OR “Sensation” OR “Sensations” OR “Pseudomelia” OR “Pseudomelia” OR “Phantom Limb Pain” OR “Phantom Limb Pains” OR “Phantom Pain” OR “Phantom Pains”)) AND (TITLE-ABS-KEY(“Transcutaneous Electric Nerve Stimulation” OR “Electric Stimulation Transcutaneous” OR “Stimulation Transcutaneous Electric” OR “Transcutaneous Electric Stimulation” OR “Percutaneous Electric Nerve Stimulation” OR “Electrical Stimulation Transcutaneous” OR “Transcutaneous Electrical Stimulation” OR “Transdermal Electrostimulation” OR “Electrostimulation Transdermal” OR “Percutaneous Electrical Nerve Stimulation” OR “Transcutaneous Electrical Nerve Stimulation” OR “Transcutaneous Nerve Stimulation” OR “Nerve Stimulation Transcutaneous” OR “Stimulation Transcutaneous Nerve” OR “TENS” OR “Percutaneous Neuromodulation Therapy” OR “Neuromodulation Therapy Percutaneous” OR “Percutaneous Neuromodulation Therapies” OR “Therapy Percutaneous Neuromodulation” OR “Percutaneous Electrical Neuromodulation” OR “Electrical Neuromodulation Percutaneous” OR “Electrical Neuromodulations Percutaneous” OR “Neuromodulation Percutaneous Electrical” OR “Neuromodulations Percutaneous Electrical” OR “Percutaneous Electrical Neuromodulations” OR “Analgesic Cutaneous Electrostimulation” OR “Cutaneous Electrostimulation Analgesic” OR “Electrostimulation Analgesic Cutaneous” OR “Electroanalgesia OR Electroanalgesias”)) AND (TITLE-ABS-KEY(“randomized controlled trial” OR“randomized” OR “placebo”))

Supplementary material 3: Studies that were evaluated in full-text and were excluded

N	Author	Year	Title	Reason for exclusion
1	Vathakul et al	2022	The Analgesic Effect of Transcutaneous Electrical Nerve Stimulation (TENS) on the	Case series
2	Mulvey et al	2013	Transcutaneous Electrical Nerve Stimulation for Phantom Pain and Stump Pain in Adult Amputees	Case series
3	Kawamura et al	1997	The Transcutaneous Electrical Nerve Stimulation Applied to Contralateral Limbs for the Phantom Limb Pain.	Case series
4	Bittar et al	2005	Deep brain stimulation for phantom limb pain	Case series
5	Pereira et al	2013	Thalamic deep brain stimulation for neuropathic pain after amputation or brachial plexus avulsion	Case series
6	Dalrymple et al	2024	A preliminary study exploring the effects of transcutaneous spinal cord stimulation on spinal excitability and phantom limb pain in people with a transtibial amputation	Case series
7	Nanivadekar et al	2023	Treatment of PostAmputation Pain With Peripheral Nerve Stimulation	Case series
8	Rauck et al	2013	Treatment of PostAmputation Pain With Peripheral Nerve Stimulation	Case series
9	Finsen et al	1988	Transcutaneous electrical nerve stimulation after major amputation	No data available
10	Abreu et al	2022	Thalamic deep brain stimulation for post-traumatic neuropathic limb pain: Efficacy at five years' follow-up and effective volume of activated brain tissue	Case series
11	Rollo et al	2011	Phantom Limb Pain: Low Frequency Repetitive Transcranial Magnetic Stimulation in Unaffected Hemisphere	Case report

12	Scibilia et al	2018	Resting-state fMR evidence of network reorganization induced by navigated transcranial magnetic repetitive stimulation in phantom limb pain	Case report
13	Grammer et al	2015	Significant Reduction in Phantom Limb Pain After Low-Frequency Repetitive Transcranial Magnetic Stimulation to the Primary Sensory Cortex	Case report
14	Pinto et al	2016	Optimizing Rehabilitation for Phantom Limb Pain Using Mirror Therapy and Transcranial Direct Current Stimulation: A Randomized, Double–Blind Clinical Trial Study Protocol	Protocol
15	Limakatso et al	2020	The effectiveness of graded motor imagery for reducing phantom limb pain in amputees: a randomised controlled trial	No data available
16	Wang et al	2024	Effects of transcranial magnetic stimulation on the human brain recorded with intracranial electrocorticography	No data available
17	Tctr et al	2022	Contralateral Transcutaneous electrical nerve stimulation (TENS) for phantom limb pain, A prospective randomized controlled trial (CTEP trial)	No data available
18	Pinto et al	2023	Detangling the Structural Neural Correlates Associated with Resting versus Dynamic Phantom Limb Pain Intensity Using a Voxel-based Morphometry Analysis	Case series
19	Pacheco-Barrios et al	2024	Using Home-based, Remotely Supervised, Transcranial Direct Current Stimulation for Phantom Limb Pain	Protocol
20	Fregni	2020	Optimizing Rehabilitation for Phantom Limb Pain Using Mirror Therapy and tDCS	Protocol
21	Kleinjung	2007	Transcranial magnetic stimulation for the treatment of auditory phantom perceptions (tinnitus) in a randomized placebo controlled study	No data available
22	Nardone	2015	Modulation of non-painful phantom,sensation in subjects with spinal cord injury by means of	No data available

			rTMS	
23	Hoffman L	2015	Management of Both Phantom Limb Pain and Residual Limb Pain with a Three-lead Spinal Cord Stimulation System	Conference abstract
24	Mills D	2016	A Case Report of Phantom Limb Pain Relief with Selective Dorsal Root Ganglion Stimulation over Spinal Cord Stimulation Following a Simultaneous Implantation Trial	Conference abstract
25	Hamani	2021	Motor cortex stimulation for chronic neuropathic pain: results of a double-blind randomized study	No data available
26	Frank, S.<br>et al	2015	Transcranial magnetic stimulation in chronic neuropathic pain-A prospective study of 45 patients	Conference abstract
27	Carroll, D.<br>et al	2000	Motor cortex stimulation for chronic neuropathic pain: a preliminary study of 10 cases	Case series
28	Boccard et al	2013	Long-term outcomes of deep brain stimulation for neuropathic pain.	Case series
29	Isagulyan, E. D.<br>et al	2011	Motor cortex stimulation is an important method in the complex treatment of central neuropathic pain	Case series
30	Pang et al	2022	Deep brain stimulation for phantom limb pain	Review
31	Lee JH et al	2015	Successful treatment of phantom limb pain by 1 Hz repetitive transcranial magnetic stimulation over affected supplementary motor complex	Case report
32	Lefaucheur<br>et al	2014	Evidence-based guidelines on the therapeutic use of repetitive transcranial magnetic stimulation (rTMS)	No data available
33	Gilmore	2019	Percutaneous peripheral nerve stimulation for the treatment of chronic neuropathic postamputation pain: a multicenter, randomized, placebo-controlled trial	No data available
34	Chamadoira<br>et al	2011	Deep brain stimulation for neuropathic pain: outcome in a series of 8 patients.	Case series

35	Roux FE	2001	Chronic motor cortex stimulation for phantom limb pain: a functional magnetic resonance imaging study: technical case report.	Case report
36	Nct,	2014	Long-term Effects of Transcranial Direct Current Stimulation (tDCS) on Patients with Phantom Limb Pain (PLP)	Protocol
37	Viswanathan et al	2010	Use of spinal cord stimulation in the treatment of phantom limb pain: case series and review of the literature.	Observational retrospective study
38	Katayama et al.	2001	Motor cortex stimulation for phantom limb pain: comprehensive therapy with spinal cord and thalamic stimulation.	Observational retrospective study
39	Eldabe et al	2015	Dorsal root ganglion (DRG) stimulation in the treatment of phantom limb pain (PLP)	Observational retrospective study
40	Eldabe et al	2015	Neurophysiology of the dorsal root ganglion (DRG): a translational premise for the use of targeted spinal cord stimulation (SCS) in the treatment of phantom limb pain (PLP)	Observational retrospective study
41	Mori	2024	Repetitive transcranial magnetic stimulation focusing on patients with neuropathic pain in the upper limb: a randomized sham-controlled parallel trial	Combined population (1 patient with PLP)

Supplementary material 3: Prism flow chart , Risk of Bias assessment, characteristics of studies

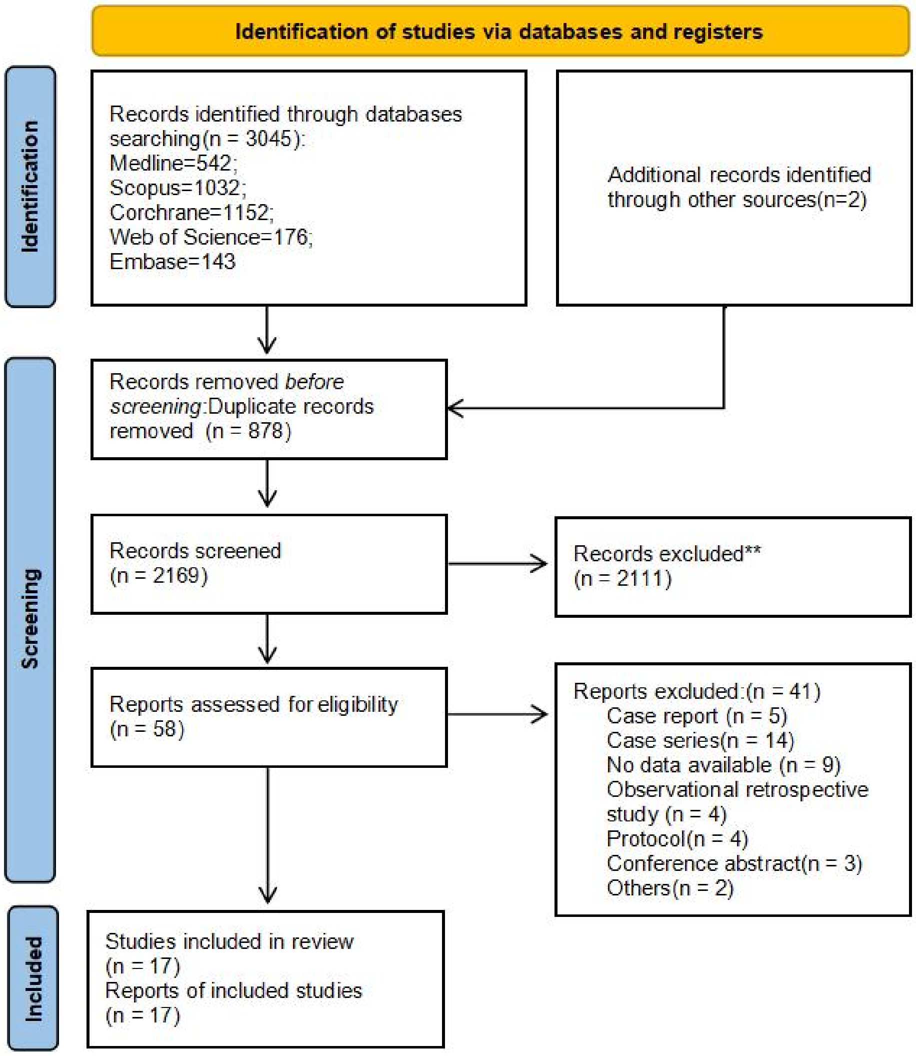

Supplementary Figure 4.1:Prism flowchart (study selection).

a

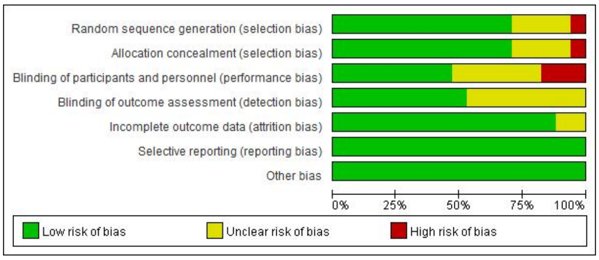

b

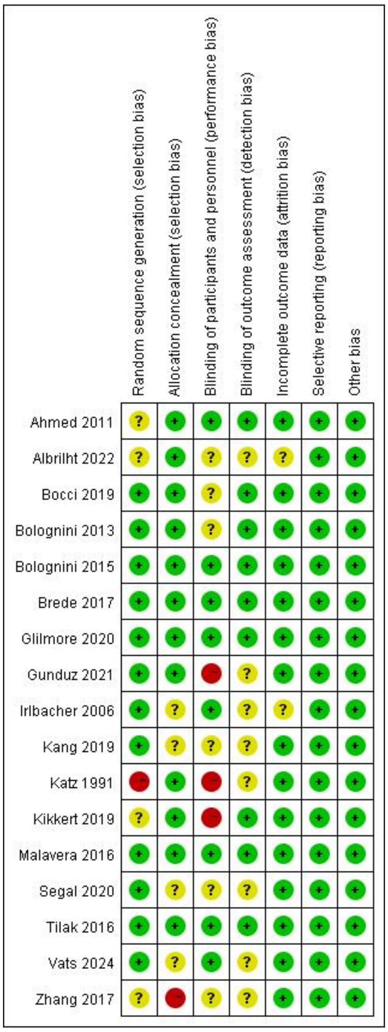

Supplementary Figure 4.2 Judgements from two reviewing authors regarding each risk of bias item presented as percentages across all included studies: (a) risk of bias graph and (b) risk of bias summary.

Supplement Table Intervention 4.3: Characteristics of studies included

First Author;Year	Design	Sample size （I/C）	Mean age （I/C）	Intervention	Stimulation Location	Control	Outcome measure and *P* Value
Ahmed 2011	RCT	27(17/10)	52±12.7 /53±13.3	**rTMS**<br>**Frequency:** 20Hz，10s trains for every minutes (200pulses)，10min sessions daily for 5 consecutive days<br>**Intensity of stimulation:** 80% of resting motor threshold	optimal scalp location from which TMS evoked motor potentials of greatest amplitude in the muscle proximal to the stump	Sham stimulation: coil elevated and angled away from the head	**VAS:** I=3.4±1.2, C=7.4±0.84 (*P*=0.001)
Malavera 2016	RCT	54(27/27)	33.1 ± 6.6 /34.7 ± 9.9	**rTMS**<br>**Frequency:** 10 Hz (1200 pulses), 20 minutes per day, during 10 days.<br>**Intensity of stimulation:** 90% of motor threshold	primary motor cortex (M1) contralateral to the amputated leg: using a figure-of-eight coil connected to a Magstim Rapid2 magnetic stimulator	sham stimulation：sham coil	**VAS:** I=2.28±2.51, C=3.71±2.97(*P*=0.03)<br>**ZSDS**：I=24.9±9.05, C=23.2±2.99(*P*＜0.05)
Irlbacher2006	RCT	28(14/14)	46.6±12.6	**rTMS**<br>Frequency:5HZ,(500 pulses), 15 minutes per day, during 5 day/week<br>**Treatment period**：for 2 weeks.<br>Intensity of stimulation: 95%	M1 area corresponding to affected phantom limb. Optimal placement defined by maximal motor response	Sham stimulation	**VAS**:<br>I= 4.99±2.33, C=4.37±2.97 (*P*=0.02)
Vats et al 2024	RCT	19(10/9)	28.87±7.98 /37±13.24	**rTMS**<br>**Frequency:**1Hz (1200 pulses), and an intertrain interval of 60 seconds, 10 sessions for 26 minutes<br>**Treatment period**：2 weeks treatment, 60 days.follow up.<br>**Intensity of stimulation:** 90% of motor threshold	surface electrodeswere placed on the abductor pollicis brevis (contralateral to the site of amputation) with a ground electrode on the wrist to acquire an electromyogram	Sham rTMS	**VAS**:I=0.19±0.24, C=3.47+-0.87 (*P*＜0.01)

Supplement Table 4.3:Continued

First Author;Year	Design	Sample size (I/C)	Mean age (I/C)	Intervention	Stimulation Location	Control	Outcome measure and *P* Value
Bolognini 2013	RCT	8 (8/8)	59.0 ± 18.75	**tDCS**<br>**Frequency:**Lasted for 15 minutes (fade-in/fade-out phase = 10 s) Single treatment<br>**Intensity of stimulation:** 2 mA	the anodal electrode was placed over C3 or C4 (EEG 10/20 system) and the cathode electrode was placed over the contralateral supraorbital area.	Sham stimulation: the stimulator was turned off after 30 s	**VAS:**I=0.8±1.37, C=2.6±1.37(*P*=0.02)
Bolognini 2015	RCT	8 (8/8)	60.75±15.09	**tDCS**<br>**Frequency:**15 min each for 5days<br>**Intensity of stimulation:** The constant current was 1.5 mA, and the ramp period at the beginning and end of stimulation was 10 seconds	The anodal electrode was placed over C3 or C4 (EEG 10/20 system), while the cathode electrode was placed on the contralateral supraorbital area.	Sham stimulation: the current lasted only for 30 sec	**VAS:** I=3.3±1.24, C=4.7±1.24 (*P*=0.04)<br>**BDI:** I=11±2.8, C=14±2.8(*P*＜*0.05*)
Kikkert 2019	RCT	32(17/15)	47.0±3/46±3	**tDCS**<br>**Frequency:**20 minutes (fade-in/fade-out phases = 10 s).4 consecutive experiments with an interval of at least 1 week.<br>**Intensity of stimulation:** 1 mA	Placed the anodal electrode on the S1/M1 deletion of the hand cortex (5cm lateral to Cz, corresponding to C3/C4), and the cathodal electrode on the contralateral supraorbital region (and vice versa, for cathodic control stimulation).	Sham stimulation: electrodes were located in the intact hand S1 / M1 and contralateral supraorbital region	**VAS:** I=1.73±3.05, C=2.5±2.56 (*P*=0.01)
Gunduz 2021	RCT	55(28/27)	39.96±15.96 /42.96±12.28	**tDCS**<br>**Frequency:**10 sessions for 2 weeks, 20 minutes each session<br>**Intensity of stimulation:** 2 mA	The anodal electrode was placed on the contralateral M1 side (on the C3 or C4, 10-20 system) on the amputated side and on the cathode of the contralateral supraorbital region.	Sham stimulation: Current was only available for the first 30 seconds	**VAS:** I=3±1.48, C=4±1.52 (*P*＜*0.05*)<br>**BDI:** I=9.14±8.72, C=8.26±5.47 (*P*＞*0.05*)
Bocci 2019	RCT	14(14/14)	40.21±9.74	**tDCS**<br>**Frequency:** 20 min per day, Monday to Friday for 5 days<br>**Intensity of stimulation:** 2.0 mA	the anode was applied on the median line, 2 cm below the inion, the medial side of the lateral epiphyseum of the papilla was about 1 cm, and the cathode is above the right shoulder	sham stimulation: the current was turned on for 5s and then turned off in a ramp-shaped fashion	**VAS**: I=5.6 ± 2.1, C= 5.9 ± 2.4 (*P*＞*0.05*)
Segal 2020	RCT	20(10/10)	58.1±10.9	**tDCS +**Mirror therapy<br>**Frequency:**once a day for 2 weeks, 20minutes each treatment period<br>**Intensity of stimulation:** 1.5mA	tDCS electrodes were inserted into 5* 7cm (35 cm2) sponges soaked with saline solution (0.9 M) and placed on participants'heads based on the side of amputation	Mirror therapy/sham tDCS+Mirror Therapy	**VAS:**I=1.43±0.70,C=5.61±0.25 (*P*<0.001)

Supplement Table 4.3:Continued

First Author;Year	Design	Sample size （I/C）	Mean age （I/C）	Intervention	Stimulation Location	Control	Outcome measure and *P* Value
Katz 1991	RCT	28(9/11/8) (PLS/PLP/No-PL)	52.8	**TENS**<br>**frequency:** Pulse rate were 4 Hz. Each session for 30min, consisted of three consecutive 10 min periods. (including an initial resting baseline(Bl), bilateral ear stimulation (BES). and a final resting baseline (B2).)<br>**intensity:** ranged from 10 to 30 volts across a fixed resistance of 2000 ohms. Stimulation intensity was increased until the subjects tell a strong but tolerable sensation	TENS was delivered to the outer ears,two silver earrings clasped the subject's earlobes.	Placebo session: nonconducting leads connected the electrical stimulator and ear electrodes so there is no current.	**MPQ:** I=5.16 ± 1.05, C= 9.0 ± 2.16(*P* <0.01)
Tilak 2016	RCT	26(13/13)	36.38±9.55/ 42.62±10.69	**TENS**<br>**Frequency:** 20 min, one session of TENS for 4 consecutive day<br>**Intensity:** the intensity of current (mA) was a strong but comfortable sensation without visible muscle contraction	at the site of phantom pain	Mirror therapy	**VAS**: I=2.46 ± 1.56, C= 2.08 ± 1.62(*P*=0.003)
Zhang 2017	RCT	26(13/13)	40.6±4.16/41.3±4.52	**TENS+Mirror Therapy**<br>**Frequency(TENS)** :20HZ,continuous wave<br>**Treatment period(TENS)**： 20min*2/day ,5d/week for 6 weeks<br>**Intensity of stimulation(TENS):**Stimulation intensity was increased until the patients tell a strong but tolerable sensation	electrodes placed on the residual limb	Mirror Therapy	**VAS**:I=5.70±0.41, C=4.10±0.36, (*P*＜0.05)
Kang 2019	RCT	40(20/20)	48.04±7.55 /52.09±7.25	**TENS+Ocupational Therapy**<br>**Frequency(TENS)** :10HZ,continuous wave<br>**Treatment period(TENS)**：40min/day ,5d/week for 4 weeks<br>**Intensity of stimulation(TENS):**Stimulation intensity was increased until the patients tell a strong but tolerable sensation	electrodes placed on the residual limb/contralateral limb depend on group	Ocupational Therapy	**MPQ**: I=5.90±1.86, C=10.75±3.04, (*P*＜0.05)<br>**SDS**:I=54.45±6.61, C=60.10±6.35, (*P*＜0.05)

Supplement Table 4.3: Continued

First Author;Year	Design	Sample size （I/C）	Mean age （I/C）	Intervention	Stimulation Location	Control	Outcome measure and *P* Value
Brede 2017	RCT	44 (23/21)	25.7±5.9/ 26.1±5.9	**NMES + MARP**<br>**Frequency:** 75 min/week (15 min/session on 5 days/week)<br>**Intensity of stimulation:** the maximum voluntary contraction in strength testing	quadriceps muscles of both legs	MARP-only: using a prosthesis in the second 6 weeks	**MPQ:** I=0.8 ± 0.7, C=1.6 ± 0.7(*P* =0.005)
Albright 2022	RCT	16(8/8)	66.5±12.5/77.0±13	**PNS**<br>**Frequency** :a fixed frequency of 100 Hz(Asymmetric charge-balanced biphasic pulse train).<br>**Intensity of stimulation:**amplitudes (0–30 mA) and pulse widths (10–200 us),intensity variable that range from 0 to 100 to patients own comfort level.<br>**Treatment period**：60days	1-3 cm distant from the femoral and sciatic nerves, region of pain	Current standard medical therapy (SMT)	**BPI**:I=1.0±1.2,C=1.3±2.2 (P＜0.05)
Glilmore 2020	RCT	24(11/13)	48.3±12.3/45.0±13.2	**PNS**<br>**Frequency** :a fixed frequency of 100 Hz(Asymmetric charge-balanced biphasic pulse train), 1-30mA, 10-200µs.<br>**Intensity of stimulation:**amplitudes (0–30 mA) and pulse widths (10–200 us),intensity variable that range from 0 to 100 to patients own comfort level.<br>**Treatment period**：60days	1-2 cm distal to the inguinal crease	Sham stimulation	**BPI**:I=3.1±2.8, C=3.8±2.2 (P＜0.05)<br>**BDI**：I=4.0±4.3, C=14.0±12.6(P ＜0.05)

I = intervention; C = control; rTMS = transcranial magnetic stimulation; tDCS = transcranial direct current stimulation; TENS = transcutaneous electrical nerve stimulation; MARP = military amputee rehabilitation program; NMES = neuromuscular electrical stimulation; PLP = phantom limb pain; VAS = visual analog scale; MPQ = McGill Pain Questionnaire; BPI=Brief Pain Inventory; NRS = numeric rating scale; BDI = Beck Depression Inventory; EEG = electroencephalography; PVG = periventricular gray.

Supplementary material 5: forest plots, subgroup analysis result

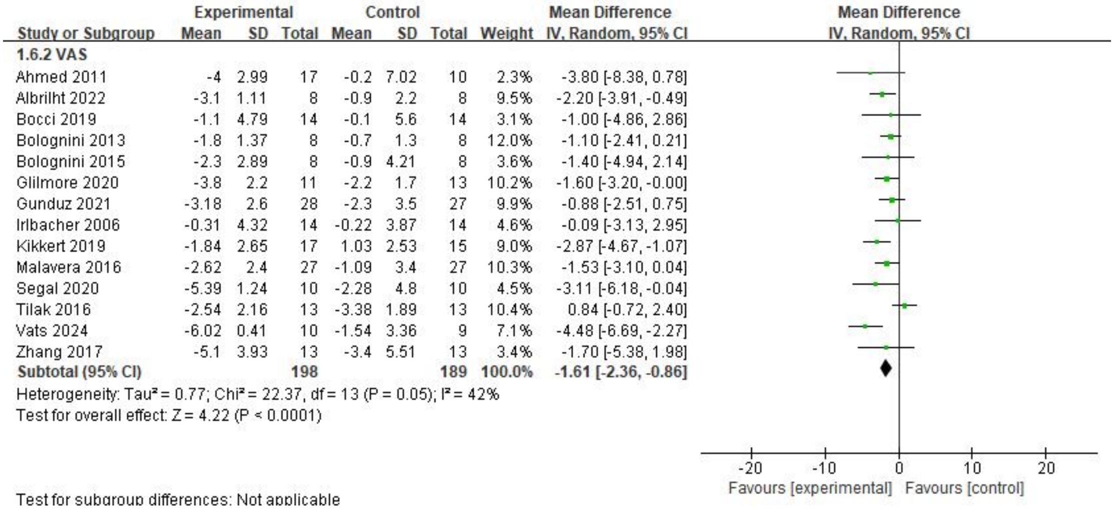

Study or Subgroup	Experimental Mean	Experimental SD	Experimental Total	Control Mean	Control SD	Control Total	Weight	Mean Difference IV, Random, 95% CI
**1.6.2 VAS**								
Ahmed 2011	-4	2.99	17	-0.2	7.02	10	2.3%	-3.80 [-8.38, 0.78]
Albrilht 2022	-3.1	1.11	8	-0.9	2.2	8	9.5%	-2.20 [-3.91, -0.49]
Bocci 2019	-1.1	4.79	14	-0.1	5.6	14	3.1%	-1.00 [-4.86, 2.86]
Bolognini 2013	-1.8	1.37	8	-0.7	1.3	8	12.0%	-1.10 [-2.41, 0.21]
Bolognini 2015	-2.3	2.89	8	-0.9	4.21	8	3.6%	-1.40 [-4.94, 2.14]
Glilmore 2020	-3.8	2.2	11	-2.2	1.7	13	10.2%	-1.60 [-3.20, -0.00]
Gunduz 2021	-3.18	2.6	28	-2.3	3.5	27	9.9%	-0.88 [-2.51, 0.75]
Irlbacher 2006	-0.31	4.32	14	-0.22	3.87	14	4.6%	-0.09 [-3.13, 2.95]
Kikkert 2019	-1.84	2.65	17	1.03	2.53	15	9.0%	-2.87 [-4.67, -1.07]
Malavera 2016	-2.62	2.4	27	-1.09	3.4	27	10.3%	-1.53 [-3.10, 0.04]
Segal 2020	-5.39	1.24	10	-2.28	4.8	10	4.5%	-3.11 [-6.18, -0.04]
Tilak 2016	-2.54	2.16	13	-3.38	1.89	13	10.4%	0.84 [-0.72, 2.40]
Vats 2024	-6.02	0.41	10	-1.54	3.36	9	7.1%	-4.48 [-6.69, -2.27]
Zhang 2017	-5.1	3.93	13	-3.4	5.51	13	3.4%	-1.70 [-5.38, 1.98]
**Subtotal (95% CI)**			**198**			**189**	**100.0%**	**-1.61 [-2.36, -0.86]**

Heterogeneity: Tau² = 0.77; Chi² = 22.37, df = 13 (P = 0.05); I² = 42%

Test for overall effect: Z = 4.22 (P < 0.0001)

Test for subgroup differences: Not applicable

Supplementary Figure 5.1 Forest plot of the effect of neuromodulation techniques on VAS index in patients with PLP

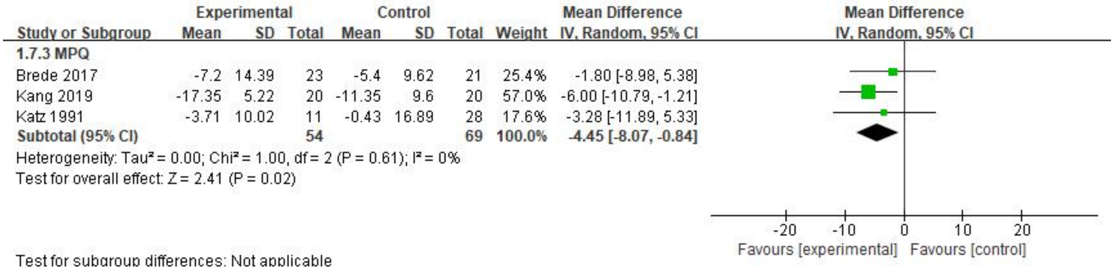

Study or Subgroup	Experimental Mean	Experimental SD	Experimental Total	Control Mean	Control SD	Control Total	Weight	Mean Difference IV, Random, 95% CI
**1.7.3 MPQ**								
Brede 2017	-7.2	14.39	23	-5.4	9.62	21	25.4%	-1.80 [-8.98, 5.38]
Kang 2019	-17.35	5.22	20	-11.35	9.6	20	57.0%	-6.00 [-10.79, -1.21]
Katz 1991	-3.71	10.02	11	-0.43	16.89	28	17.6%	-3.28 [-11.89, 5.33]
**Subtotal (95% CI)**			**54**			**69**	**100.0%**	**-4.45 [-8.07, -0.84]**

Heterogeneity: Tau² = 0.00; Chi² = 1.00, df = 2 (P = 0.61); I² = 0%

Test for overall effect: Z = 2.41 (P = 0.02)

Test for subgroup differences: Not applicable

Supplementary Figure 5.2 Forest plot of the effect of neuromodulation techniques on MPQ index in patients with PLP

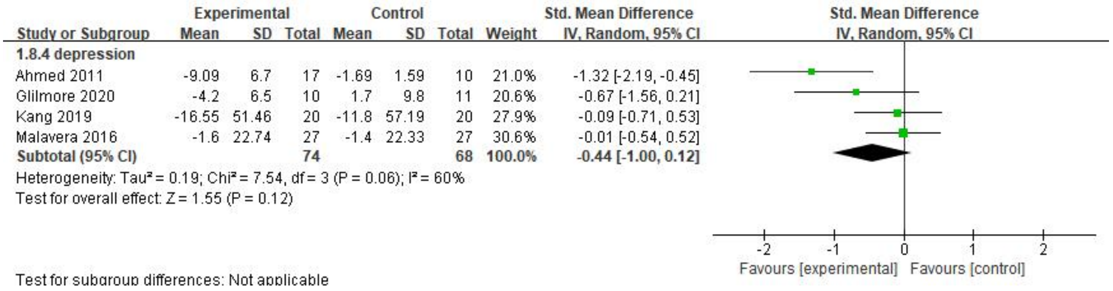

Study or Subgroup	Experimental Mean	Experimental SD	Experimental Total	Control Mean	Control SD	Control Total	Weight	Std. Mean Difference IV, Random, 95% CI
**1.8.4 depression**								
Ahmed 2011	-9.09	6.7	17	-1.69	1.59	10	21.0%	-1.32 [-2.19, -0.45]
Glilmore 2020	-4.2	6.5	10	1.7	9.8	11	20.6%	-0.67 [-1.56, 0.21]
Kang 2019	-16.55	51.46	20	-11.8	57.19	20	27.9%	-0.09 [-0.71, 0.53]
Malavera 2016	-1.6	22.74	27	-1.4	22.33	27	30.6%	-0.01 [-0.54, 0.52]
**Subtotal (95% CI)**			**74**			**68**	**100.0%**	**-0.44 [-1.00, 0.12]**

Heterogeneity: Tau² = 0.19; Chi² = 7.54, df = 3 (P = 0.06); I² = 60%

Test for overall effect: Z = 1.55 (P = 0.12)

Test for subgroup differences: Not applicable

Supplementary Figure 5.3 Forest plot of the effect of neuromodulation techniques on Depression index in patients with PLP

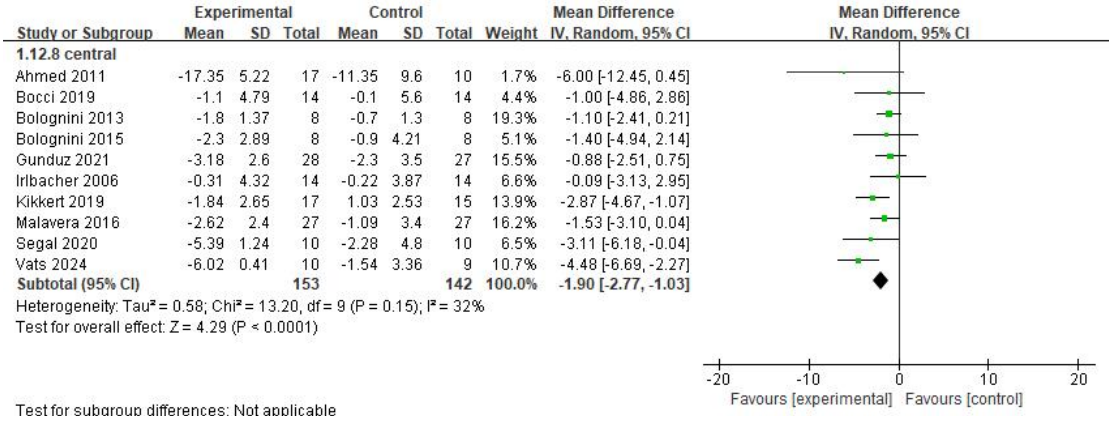

Supplementary Figure 5.4 Forest plot of the effect of neuromodulation techniques on Central index in patients with PLP

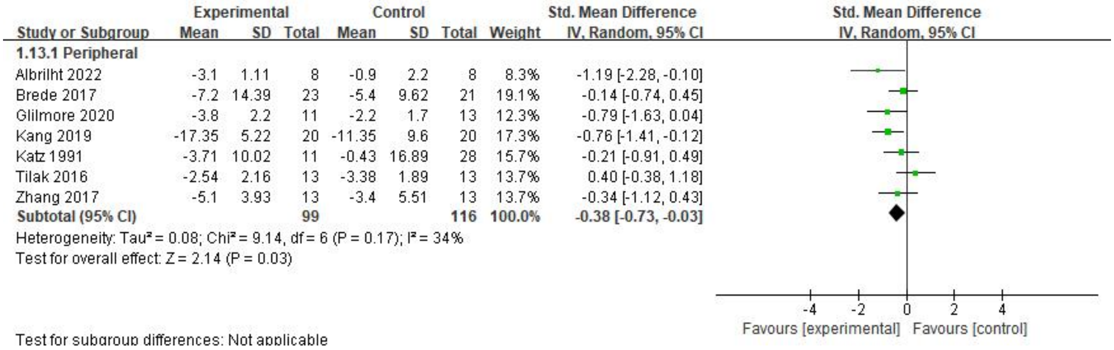

Supplementary Figure 5.5 Forest plot of the effect of neuromodulation techniques on peripheral index in patients with PLP

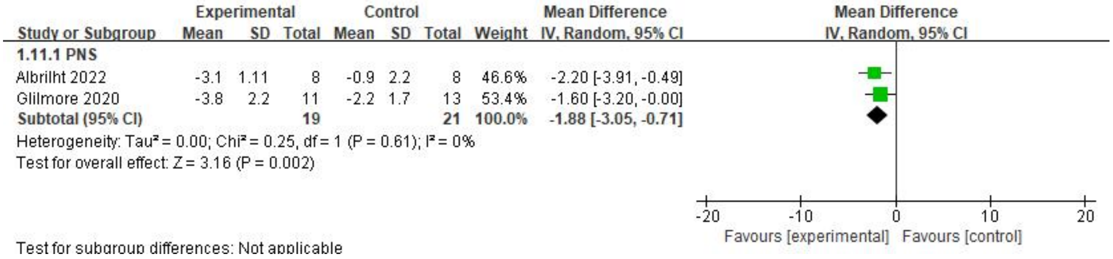

Supplementary Figure 5.6 Forest plot of the effect of neuromodulation techniques on PNS index in patients with PLP

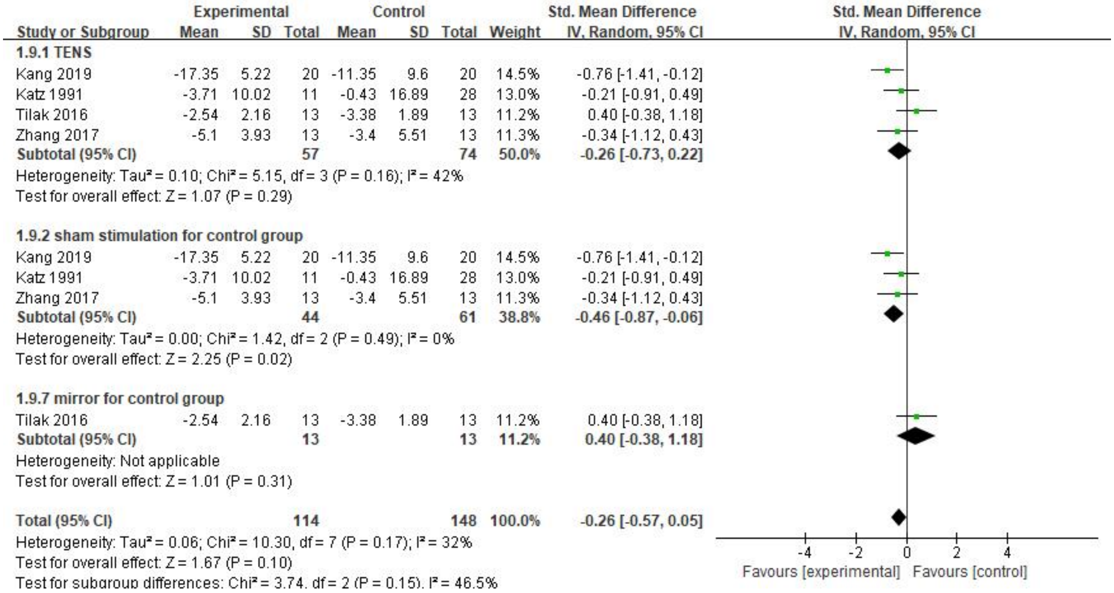

Study or Subgroup	Experimental Mean	Experimental SD	Experimental Total	Control Mean	Control SD	Control Total	Weight	Std. Mean Difference IV, Random, 95% CI
**1.9.1 TENS**								
Kang 2019	-17.35	5.22	20	-11.35	9.6	20	14.5%	-0.76 [-1.41, -0.12]
Katz 1991	-3.71	10.02	11	-0.43	16.89	28	13.0%	-0.21 [-0.91, 0.49]
Tilak 2016	-2.54	2.16	13	-3.38	1.89	13	11.2%	0.40 [-0.38, 1.18]
Zhang 2017	-5.1	3.93	13	-3.4	5.51	13	11.3%	-0.34 [-1.12, 0.43]
**Subtotal (95% CI)**			**57**			**74**	**50.0%**	**-0.26 [-0.73, 0.22]**
Heterogeneity: Tau² = 0.10; Chi² = 5.15, df = 3 (P = 0.16); I² = 42%								
Test for overall effect: Z = 1.07 (P = 0.29)								
**1.9.2 sham stimulation for control group**								
Kang 2019	-17.35	5.22	20	-11.35	9.6	20	14.5%	-0.76 [-1.41, -0.12]
Katz 1991	-3.71	10.02	11	-0.43	16.89	28	13.0%	-0.21 [-0.91, 0.49]
Zhang 2017	-5.1	3.93	13	-3.4	5.51	13	11.3%	-0.34 [-1.12, 0.43]
**Subtotal (95% CI)**			**44**			**61**	**38.8%**	**-0.46 [-0.87, -0.06]**
Heterogeneity: Tau² = 0.00; Chi² = 1.42, df = 2 (P = 0.49); I² = 0%								
Test for overall effect: Z = 2.25 (P = 0.02)								
**1.9.7 mirror for control group**								
Tilak 2016	-2.54	2.16	13	-3.38	1.89	13	11.2%	0.40 [-0.38, 1.18]
**Subtotal (95% CI)**			**13**			**13**	**11.2%**	**0.40 [-0.38, 1.18]**
Heterogeneity: Not applicable								
Test for overall effect: Z = 1.01 (P = 0.31)								
**Total (95% CI)**			**114**			**148**	**100.0%**	**-0.26 [-0.57, 0.05]**
Heterogeneity: Tau² = 0.06; Chi² = 10.30, df = 7 (P = 0.17); I² = 32%								
Test for overall effect: Z = 1.67 (P = 0.10)								
Test for subgroup differences: Chi² = 3.74, df = 2 (P = 0.15), I² = 46.5%								

Supplementary Figure 5.7 Forest plot of the effect of neuromodulation techniques on TENS index in patients with PLP

Study or Subgroup	Experimental Mean	Experimental SD	Experimental Total	Control Mean	Control SD	Control Total	Weight	Mean Difference IV, Random, 95% CI
**1.17.1 Anodal M1**								
Bolognini 2013	-1.8	1.37	8	-0.7	1.3	8	8.1%	-1.10 [-2.41, 0.21]
Bolognini 2015	-2.3	2.89	8	-0.9	4.21	8	1.1%	-1.40 [-4.94, 2.14]
Gunduz 2021	-3.18	2.6	28	-2.3	3.5	27	5.2%	-0.88 [-2.51, 0.75]
Kikkert 2019	-1.84	2.65	17	1.03	2.53	15	4.3%	-2.87 [-4.67, -1.07]
Segal 2020	-5.39	1.24	10	-2.28	4.8	10	1.5%	-3.11 [-6.18, -0.04]
**Subtotal (95% CI)**			**71**			**68**	**20.0%**	**-1.60 [-2.45, -0.74]**
Heterogeneity: Tau² = 0.04; Chi² = 4.17, df = 4 (P = 0.38); I² = 4%								
Test for overall effect: Z = 3.66 (P = 0.0003)								
**1.17.2 Anodal PPC**								
Bolognini 2013	-1.8	1.37	8	-0.7	1.3	8	8.1%	-1.10 [-2.41, 0.21]
**Subtotal (95% CI)**			**8**			**8**	**8.1%**	**-1.10 [-2.41, 0.21]**
Heterogeneity: Not applicable								
Test for overall effect: Z = 1.65 (P = 0.10)								
**1.17.3 Anodal Cerebellum**								
Bocci 2019	-1.1	4.79	14	-0.1	5.6	14	0.9%	-1.00 [-4.86, 2.86]
**Subtotal (95% CI)**			**14**			**14**	**0.9%**	**-1.00 [-4.86, 2.86]**
Heterogeneity: Not applicable								
Test for overall effect: Z = 0.51 (P = 0.61)								
**1.17.4 Cathodal PPC**								
Bolognini 2013	-1.8	1.37	8	-0.7	1.3	8	8.1%	-1.10 [-2.41, 0.21]
**Subtotal (95% CI)**			**8**			**8**	**8.1%**	**-1.10 [-2.41, 0.21]**
Heterogeneity: Not applicable								
Test for overall effect: Z = 1.65 (P = 0.10)								
**1.17.5 tDCS >7 days**								
Gunduz 2021	-3.18	2.6	28	-2.3	3.5	27	5.2%	-0.88 [-2.51, 0.75]
Segal 2020	-5.39	1.24	10	-2.28	4.8	10	1.5%	-3.11 [-6.18, -0.04]
**Subtotal (95% CI)**			**38**			**37**	**6.6%**	**-1.60 [-3.64, 0.44]**
Heterogeneity: Tau² = 0.91; Chi² = 1.58, df = 1 (P = 0.21); I² = 37%								
Test for overall effect: Z = 1.53 (P = 0.12)								
**1.17.10 tDCS ⩽7 days**								
Bocci 2019	-1.1	4.79	14	-0.1	5.6	14	0.9%	-1.00 [-4.86, 2.86]
Bolognini 2013	-1.8	1.37	8	-0.7	1.3	8	8.1%	-1.10 [-2.41, 0.21]
Bolognini 2015	-2.3	2.89	8	-0.9	4.21	8	1.1%	-1.40 [-4.94, 2.14]
Kikkert 2019	-1.84	2.65	17	1.03	2.53	15	4.3%	-2.87 [-4.67, -1.07]
**Subtotal (95% CI)**			**47**			**45**	**14.4%**	**-1.64 [-2.62, -0.66]**
Heterogeneity: Tau² = 0.00; Chi² = 2.58, df = 3 (P = 0.46); I² = 0%								
Test for overall effect: Z = 3.29 (P = 0.001)								
**1.17.12 tDCS ⩽1.5mA**								
Bolognini 2015	-2.3	2.89	8	-0.9	4.21	8	1.1%	-1.40 [-4.94, 2.14]
Kikkert 2019	-1.84	2.65	17	1.03	2.53	15	4.3%	-2.87 [-4.67, -1.07]
Segal 2020	-5.39	1.24	10	-2.28	4.8	10	1.5%	-3.11 [-6.18, -0.04]
**Subtotal (95% CI)**			**35**			**33**	**6.8%**	**-2.68 [-4.10, -1.26]**
Heterogeneity: Tau² = 0.00; Chi² = 0.62, df = 2 (P = 0.73); I² = 0%								
Test for overall effect: Z = 3.70 (P = 0.0002)								
**1.17.13 tDCS >1.5mA**								
Bocci 2019	-1.1	4.79	14	-0.1	5.6	14	0.9%	-1.00 [-4.86, 2.86]
Bolognini 2013	-1.8	1.37	8	-0.7	1.3	8	8.1%	-1.10 [-2.41, 0.21]
Gunduz 2021	-3.18	2.6	28	-2.3	3.5	27	5.2%	-0.88 [-2.51, 0.75]
**Subtotal (95% CI)**			**50**			**49**	**14.1%**	**-1.01 [-2.00, -0.03]**
Heterogeneity: Tau² = 0.00; Chi² = 0.04, df = 2 (P = 0.98); I² = 0%								
Test for overall effect: Z = 2.01 (P = 0.04)								
**1.17.14 tDCS ⩽15min**								
Bolognini 2013	-1.8	1.37	8	-0.7	1.3	8	8.1%	-1.10 [-2.41, 0.21]
Bolognini 2015	-2.3	2.89	8	-0.9	4.21	8	1.1%	-1.40 [-4.94, 2.14]
**Subtotal (95% CI)**			**16**			**16**	**9.2%**	**-1.14 [-2.36, 0.09]**
Heterogeneity: Tau² = 0.00; Chi² = 0.02, df = 1 (P = 0.88); I² = 0%								
Test for overall effect: Z = 1.81 (P = 0.07)								
**1.17.15 tDCS >15min**								
Bocci 2019	-1.1	4.79	14	-0.1	5.6	14	0.9%	-1.00 [-4.86, 2.86]
Gunduz 2021	-3.18	2.6	28	-2.3	3.5	27	5.2%	-0.88 [-2.51, 0.75]
Kikkert 2019	-1.84	2.65	17	1.03	2.53	15	4.3%	-2.87 [-4.67, -1.07]
Segal 2020	-5.39	1.24	10	-2.28	4.8	10	1.5%	-3.11 [-6.18, -0.04]
**Subtotal (95% CI)**			**69**			**66**	**11.8%**	**-1.91 [-3.10, -0.72]**
Heterogeneity: Tau² = 0.19; Chi² = 3.42, df = 3 (P = 0.33); I² = 12%								
Test for overall effect: Z = 3.15 (P = 0.002)								
**Total (95% CI)**			**356**			**344**	**100.0%**	**-1.48 [-1.86, -1.11]**
Heterogeneity: Tau² = 0.00; Chi² = 17.79, df = 25 (P = 0.85); I² = 0%								
Test for overall effect: Z = 7.83 (P < 0.00001)								
Test for subgroup differences: Chi² = 5.32, df = 9 (P = 0.81), I² = 0%								

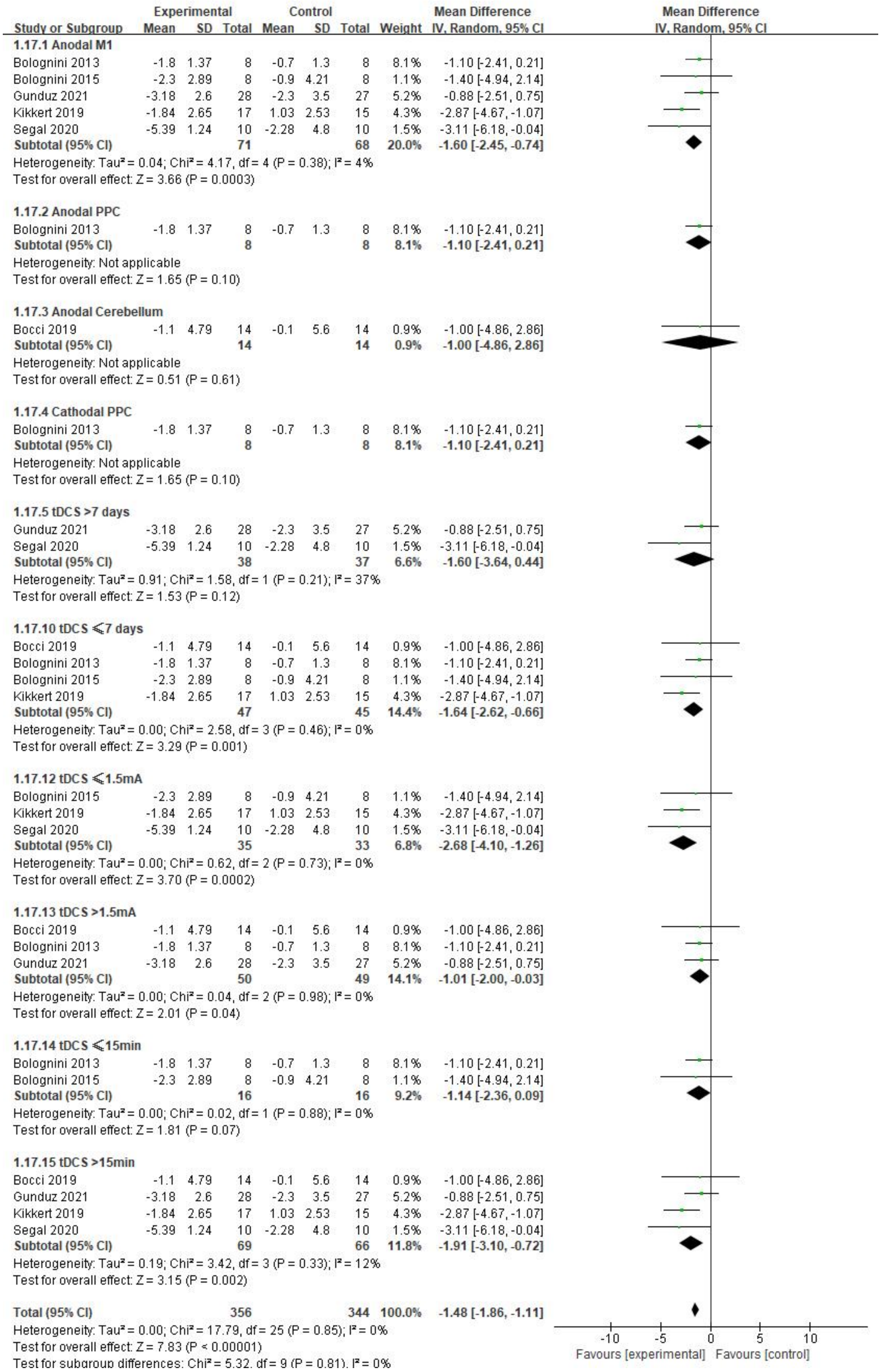

Supplementary Figure 5.8 Forest plot of different types and parameters of tDCS

Study or Subgroup	Experimental Mean	Experimental SD	Experimental Total	Control Mean	Control SD	Control Total	Weight	Mean Difference IV, Random, 95% CI
**1.16.1 Excitatory M1**								
Ahmed 2011	-4	2.99	17	-0.2	7.02	10	3.8%	-3.80 [-8.38, 0.78]
Irlbacher 2006	-0.31	4.32	14	-0.22	3.87	14	6.8%	-0.09 [-3.13, 2.95]
Malavera 2016	-2.62	2.4	27	-1.09	3.4	27	12.9%	-1.53 [-3.10, 0.04]
**Subtotal (95% CI)**			**58**			**51**	**23.5%**	**-1.45 [-2.78, -0.11]**
Heterogeneity: Tau² = 0.00; Chi² = 1.79, df = 2 (P = 0.41); I² = 0%								
Test for overall effect: Z = 2.12 (P = 0.03)								
**1.16.2 Inhibitory DLPFC**								
Vats 2024	-6.02	0.41	10	-1.54	3.36	9	9.8%	-4.48 [-6.69, -2.27]
**Subtotal (95% CI)**			**10**			**9**	**9.8%**	**-4.48 [-6.69, -2.27]**
Heterogeneity: Not applicable								
Test for overall effect: Z = 3.97 (P < 0.0001)								
**1.16.4 rTMS ⩾7 days**								
Ahmed 2011	-4	2.99	17	-0.2	7.02	10	3.8%	-3.80 [-8.38, 0.78]
Vats 2024	-6.02	0.41	10	-1.54	3.36	9	9.8%	-4.48 [-6.69, -2.27]
**Subtotal (95% CI)**			**27**			**19**	**13.6%**	**-4.35 [-6.34, -2.36]**
Heterogeneity: Tau² = 0.00; Chi² = 0.07, df = 1 (P = 0.79); I² = 0%								
Test for overall effect: Z = 4.29 (P < 0.0001)								
**1.16.5 rTMS<7days**								
Irlbacher 2006	-0.31	4.32	14	-0.22	3.87	14	6.8%	-0.09 [-3.13, 2.95]
Malavera 2016	-2.62	2.4	27	-1.09	3.4	27	12.9%	-1.53 [-3.10, 0.04]
**Subtotal (95% CI)**			**41**			**41**	**19.8%**	**-1.23 [-2.62, 0.17]**
Heterogeneity: Tau² = 0.00; Chi² = 0.68, df = 1 (P = 0.41); I² = 0%								
Test for overall effect: Z = 1.72 (P = 0.08)								
**1.16.18 rTMS ⩽20min**								
Ahmed 2011	-4	2.99	17	-0.2	7.02	10	3.8%	-3.80 [-8.38, 0.78]
Irlbacher 2006	-0.31	4.32	14	-0.22	3.87	14	6.8%	-0.09 [-3.13, 2.95]
Malavera 2016	-2.62	2.4	27	-1.09	3.4	27	12.9%	-1.53 [-3.10, 0.04]
**Subtotal (95% CI)**			**58**			**51**	**23.5%**	**-1.45 [-2.78, -0.11]**
Heterogeneity: Tau² = 0.00; Chi² = 1.79, df = 2 (P = 0.41); I² = 0%								
Test for overall effect: Z = 2.12 (P = 0.03)								
**1.16.19 rTMS >20min**								
Vats 2024	-6.02	0.41	10	-1.54	3.36	9	9.8%	-4.48 [-6.69, -2.27]
**Subtotal (95% CI)**			**10**			**9**	**9.8%**	**-4.48 [-6.69, -2.27]**
Heterogeneity: Not applicable								
Test for overall effect: Z = 3.97 (P < 0.0001)								
**Total (95% CI)**			**204**			**180**	**100.0%**	**-2.36 [-3.35, -1.37]**
Heterogeneity: Tau² = 1.33; Chi² = 21.31, df = 11 (P = 0.03); I² = 48%								
Test for overall effect: Z = 4.67 (P < 0.00001)								
Test for subgroup differences: Chi² = 16.97, df = 5 (P = 0.005), I² = 70.5%								

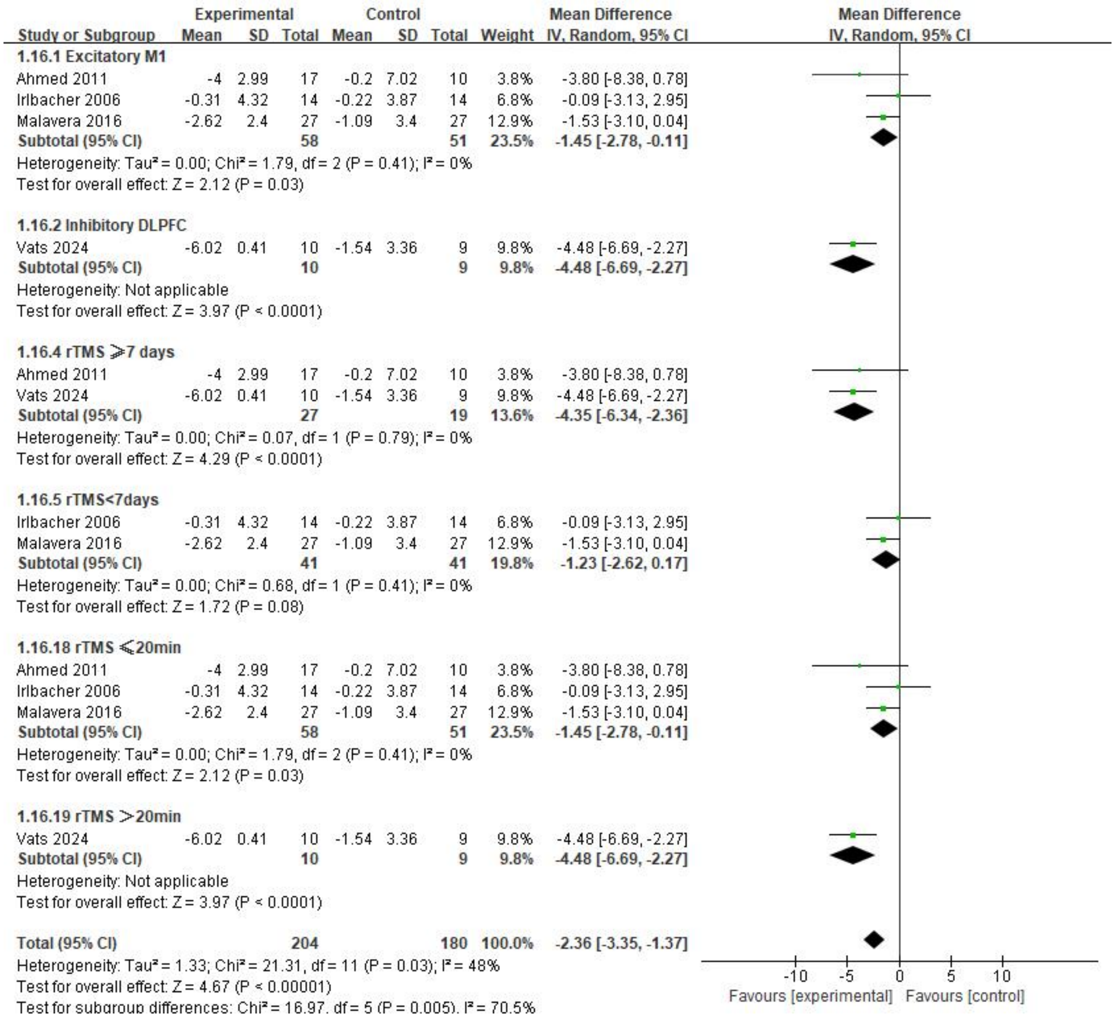

Supplementary Figure 5.9 Forest plot of different types and parameters of rTMS

Supplementary material 6: funnel plots, sensitivity analysis result

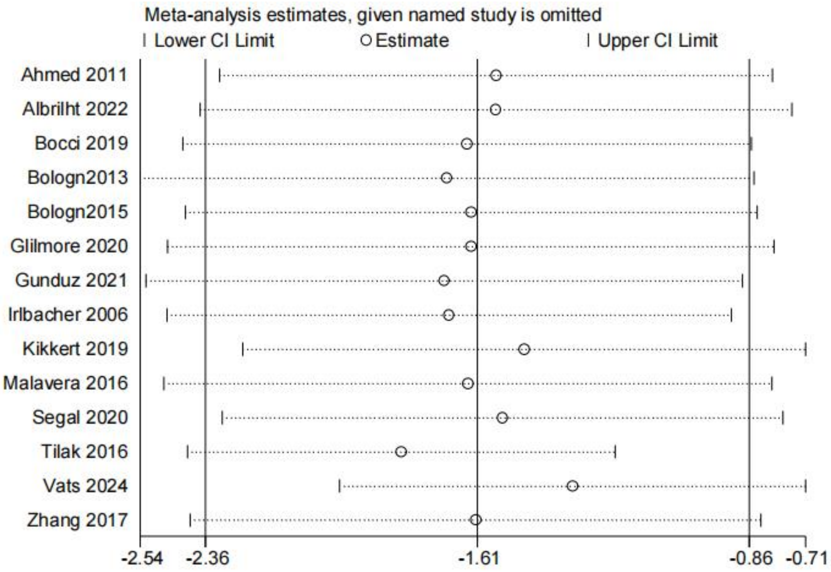

Supplementary Figure 6.1 Sensitivity analysis of VAS

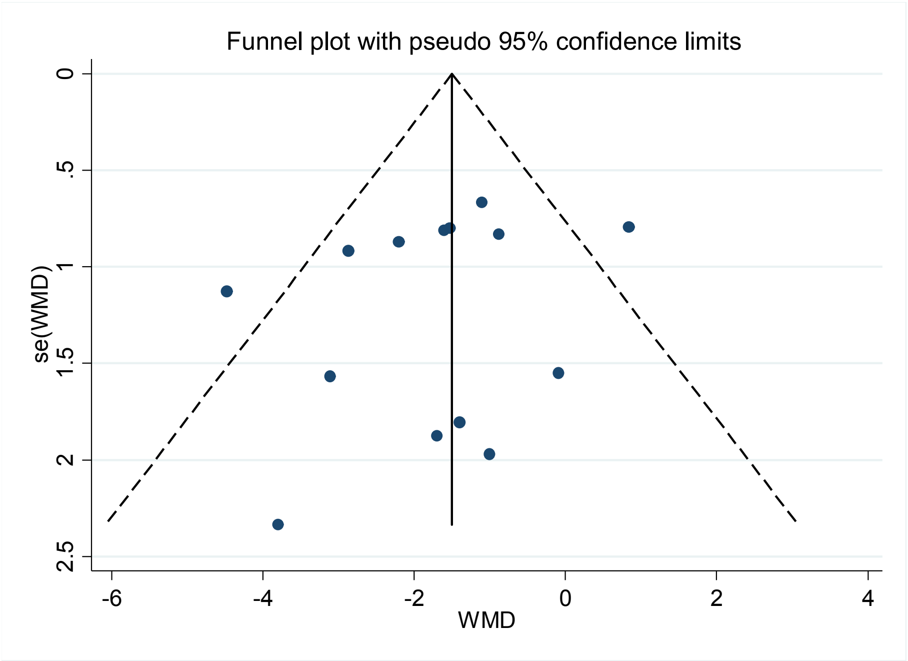

Supplementary Figure 6.2 Funnel plot of the meta-analysis for VAS.

Number of studies = **14** Root MSE = **1.312**

Std_Eff	Coef.	Std. Err.	t	P>|t|	[95% Conf.	Interval]
slope	-.5498546	1.017896	-0.54	0.599	-2.76766	1.667951
bias	-.9957097	.9987397	-1.00	0.338	-3.171777	1.180357

Test of H0: no small-study effects P = **0.338**

Supplementary Figure 6.3 Egger’s test for VAS

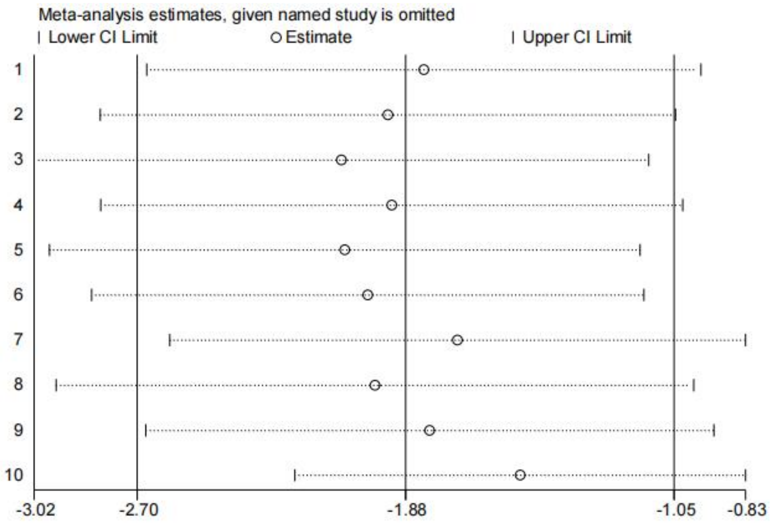

Supplementary Figure 6.4 Sensitivity analysis of Central

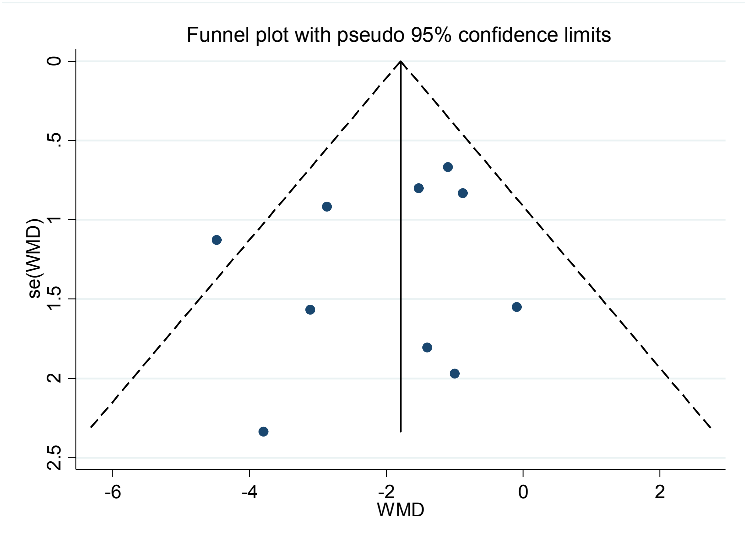

Supplementary Figure 6.5 Funnel plot of the meta-analysis for Central.

```
Egger's test for small-study effects:
Regress standard normal deviate of intervention
effect estimate against its standard error

Number of studies =   10                              Root MSE      =    1.069
```

Std_Eff	Coef.	Std. Err.	t	P>|t|	[95% Conf.	Interval]
slope	.3917822	.5348233	0.73	0.485	-.8415224	1.625087
bias	-2.586965	1.399742	-1.85	0.102	-5.814775	.6408461

```
Test of H0: no small-study effects          P = 0.102
```

Supplementary Figure 6.6 Egger’s test for Central

Supplementary material 7: subgroup analysis result

Supplementary Table 1 Subgroup analysis of the efficacy of different treatment modalities and treatment type

Subgroup	Studies/Sample	Heterogeneity test results		Effect model	Meta-analysis results	
		P	I^2（%）		MD/SMD(95%CI)	*P*
Method						
rTMS	4/128	0.07	58	Random	-2.37 [-4.35, -0.39]	0.02
tDCS	6/167	0.51	0	Random	-1.56 [-2.37, -0.75]	0.0002
PNS	2/40	0.61	0	Random	-1.88 [-3.05, -0.71]	0.002
TENS	4/131	0.16	42	Random	-0.26 [-0.73, 0.22]	0.29
Control group (TENS)						
VS Sham stimulation	3/105	0.49	0	Random	-0.46 [-0.87, -0.06]	0.02
VS Mirror therapy	1/26	NA	NA	Random	0.40 [-0.38, 1.18]	0.31
Type						
Central	10/295	0.21	25	Random	-0.55 [-0.79, -0.32]	＜0.00001
Peripheral	7/215	0.17	34	Random	-0.38 [-0.73, -0.03]	0.03

Supplementary Table 2 Subgroup analysis of the efficacy of tDCS with different treatment conditions in PLP patients

Subgroup	Studies(Sample)	Heterogeneity test results		Effect model	Meta-analysis results	
		P	I^2（%）		MD/SMD(95%CI)	*P*
Intensity						
＞1.5mA	3/99	0.98	0	Random	-1.01 [-2.00, -0.03]	0.04
≤1.5mA	3/68	0.73	0	Random	-2.68 [-4.10, -1.26]	0.0002
Duration						
＞15min	4/135	0.33	12	Random	-1.91 [-3.10, -0.72]	0.002
≤15min	2/32	0.88	0	Random	-1.14 [-2.36, 0.09]	0.07
Period						
≥1week	2/75	0.21	37	Random	-1.60 [-3.64, 0.44]	0.12
<1week	4/92	0.46	0	Random	-1.60 [-2.58, -0.62]	0.001

Supplementary Table 3 Subgroup analysis of the efficacy of rTMS with different treatment conditions in PLP patients2

Subgroup	Studies(Sample)	Heterogeneity test results		Effect model	Meta-analysis results	
		P	I^2（%）		MD/SMD(95%CI)	*P*
Intensity						
＞1HZ	3/109	0.51	0	Random	-1.48 [-2.81, -0.15]	0.03
≤1HZ	1/19	NA	NA	NA	-4.48 [-6.69, -2.27]	＜0.0001
Duration						
＞20min	1/19	NA	NA	NA	-4.48 [-6.69, -2.27]	＜0.0001
≤20min	3/133	0.41	0	Random	-1.45 [-2.78, -0.11]	0.03
Period						
≥1week	2/46	0.79	0	Random	-4.35 [-6.34, -2.36]	＜0.0001
＜1week	2/82	0.41	0	Random	-1.23 [-2.62, 0.17]	0.08
